# Tethered Catalytic Hairpin Assembly with Plasmon‐Enhanced Fluorescence Readout for Single Molecule Detection

**DOI:** 10.1002/smtd.202500037

**Published:** 2025-04-10

**Authors:** Naoto Asai, Katharina Schmidt, Gizem Aktuğ, Stefan Fossati, Juraj Sladek, N. Scott Lynn, Jakub Dostalek

**Affiliations:** ^1^ LiST – Laboratory for Life Sciences and Technology Danube Private University Viktor Kaplan‐Straße 2 Wiener Neustadt 2700 Austria; ^2^ FZU‐Institute of Physics Czech Academy of Sciences Na Slovance 2 Prague 182 21 Czech Republic; ^3^ Faculty of Mathematics and Physics Charles University Prague 121 16 Czech Republic

**Keywords:** catalytic hairpin assembly, flexible DNA linker, plasmon‐enhanced fluorescence, sandwich immunoassay, single molecule detection

## Abstract

Here a novel digital bioassay readout concept is reported that does not rely on enzymatic amplification nor compartmenting of an analyzed liquid sample. Rather, it is based on counting individual affinity‐captured target biomolecules via the use of a tethered catalytic hairpin assembly (tCHA) deployed on a solid sensor surface with spatial confinement utilized by a flexible polymer linker (FPL). Wide‐field plasmon‐enhanced fluorescence (PEF) imaging is employed for optical real‐time probing of the reaction kinetics, where affinity‐captured target molecules are manifested as spatially distinct bright fluorescent spots. The effect of the length of the FPLs is investigated, and the analytical performance of the dual amplification tCHA‐PEF concept is tested by using a model short single‐stranded DNA analyte. When applied in a sandwich immunoassay, the detection of target proteins at sub‐femtomolar concentrations is demonstrated. The reported experiments are supported by diffusion‐limited mass transfer models and document the potential of tCHA‐PEF as a new class of generic enzyme‐free bioanalytical tools enabling the ultrasensitive analysis of trace amounts of protein and nucleic acid analytes, making it attractive for future molecular diagnostics and research applications.

## Graphical abstract

This work presents a single‐molecule detection platform that does not rely on the partitioning of analyzed liquid samples and on enzymatic amplification of fluorescence signals. A flexible polymer linker serves to spatially confine catalytic‐harpin assembly‐based DNA amplification. In conjunction with a plasmon‐enhanced fluorescence readout, this versatile approach allows for ultrasensitive analysis of oligonucleotide and protein analytes.

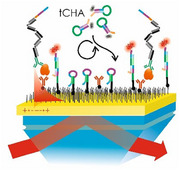

## 1 Introduction

Bioassays with a digital readout format have become an established approach for the ultra‐sensitive analysis of many types of biomolecules used in biomarker analysis, including nucleic acids (digital polymerase chain reaction – dPCR)^[^ ^1^ ^]^ and proteins (digital enzyme‐linked immunosorbent assays – dELISA).^[^ ^2^ ^]^ These methods allow for single molecule detection (SMD), with a goal of counting individual target molecules present in an analyzed liquid sample that is partitioned into a series of miniature compartments (microwells or droplets).^[^ ^3^ ^]^ The loading of target molecules into such compartments is governed by Poisson statistics,^[^ ^4^ ^]^ where detection is based on target‐induced enzymatic amplification.^[^ ^5^, ^6^ ^]^ Most commonly, a fluorescence output signal is generated in each compartment (serving as a reaction chamber) by using polymerases in combination with fluorescent oligos or alternatively, by using β‐galactosidase labels reacting with a fluorescence substrate (other approaches, e.g. catalytic microbubble amplification,^[^ ^7^ ^]^ have been also demonstrated). In general, the need to partition the analyzed sample introduces inherent statistical variability associated with uneven target distribution across compartments, which complicates quantitative analysis.^[^ ^8^ ^]^ Moreover, the use of enzymatic amplification limits the ability to deploy and store the assay constituents outside specialized laboratories and can be prone to errors due to enzyme degradation or suboptimal reaction conditions.^[^ ^9^ ^]^ Therefore, such reliance on both enzymes and sample compartmenting (both complex processes) lowers the robustness and reproducibility of digital assays, especially in non‐laboratory settings.

Simplified digital assay formats that do not rely on physical partitioning of the analyzed sample have been conceived using surface‐attached biorecognition molecules that are specific to the target analyte and can serve as individual sensing elements. The detection of target analyte that is affinity‐captured from the analyzed liquid sample can be carried out at the single molecule level by using short DNA tags: in conjunction with an isothermal amplification step, these tags can be prolonged to form long DNA chains carrying a high number of repeating motives for subsequent post‐labeling with fluorescent emitters conjugated with short complementary oligos. Previous reports on such DNA amplification include those based on polymerase‐based rolling circle amplification (RCA),^[^ ^10^, ^11^ ^]^ hybridization chain reaction (HCR),^[^ ^12^ ^]^ and catalytic hairpin assembly (CHA),^[^ ^13^ ^]^ where the post‐labeling step with fluorophore emitters allow for the identification of single target molecules manifested as spatially separated bright fluorescent spots. Thanks to previously established protocols, isothermal DNA amplification has been employed in numerous ultrasensitive bioassays for nucleic acids,^[^ ^14^ ^]^ proteins,^[^ ^15^ ^]^ and extracellular vesicles.^[^ ^16^ ^]^ However, translating research in these compartment‐free digital assays to applications in important bioanalytical fields (e.g., biomarker analysis) is still impeded by the necessity to use enzymes (such as in RCA), long amplification time with low efficiency (often observed in HCR), and additional technical drawbacks such the reliance on high‐end confocal microscopes.^[^ ^17^ ^]^ In order to address these limitations, several groups have pursued assays with other types of labeling that can resolve individual binding events, including up‐converting fluorescent nanoparticles to decrease a fluorescence background,^[^ ^18^ ^]^ or by using fluorescence amplification via photonic crystal structures that enable the imaging of individual quantum dot labels.^[^ ^19^ ^]^

Herein, we report on an alternative digital readout assay that overcomes the use of enzymes and sample partitioning. It is based on CHA, a robust non‐enzymatic DNA amplification method gaining increasing interest in the bioanalytical community.^[^ ^20^, ^21^, ^22^ ^]^ It further relies on isothermal toehold‐mediated strand displacement (TMSD), which has been successfully applied for the analysis of low concentrations of intracellular nucleic acid‐based analytes in the ensemble regime.^[^ ^23^, ^24^ ^]^ Moreover, CHA has been applied in SMD systems utilizing microdroplet‐based partitioning,^[^ ^25^ ^]^ spatial discrimination of an amplification reaction on individual colloidal metallic nanoparticles with quenching modulation,^[^ ^13^ ^]^ colloidal metallic nanoparticles with switchable quadruplex motives for docking of emitters using a plasmonic enhanced fluorescence readout,^[^ ^26^ ^]^ and by employing the detection of CHA fluorescent products at single emitter level when immobilized on a solid sensor surface.^[^ ^27^ ^]^ When tailored for SMD, these previous works rely on the recycling of the target analyte that is allowed to diffuse in the analyzed solution. Therefore, the detected fluorescent features can become spatially smeared, which complicates their association with the presence of individual target molecules.

This work reports on a new CHA‐based SMD approach that allows for the direct identification of target molecules by using a flexible polymer linker ‐ FPL‐ in conjunction with plasmon‐enhanced fluorescence (PEF) detection. The FPL serves to spatially confine both the initiator probe and CHA to locations where the target analyte molecules are affinity captured on the sensor surface. Such tethered catalytic hairpin assembly (tCHA) takes advantage of PEF, which provides an additional optical enhancement of fluorescence signal from photoactive compounds present close to the sensor surface.^[^ ^28^, ^29^, ^30^ ^]^ This allows for the real‐time wide‐field fluorescence microscopy‐based monitoring of the amplification reaction on the sensor surface that is not masked by the parasitic fluorescence signal originating from assay constituents dissolved in the contacted bulk aqueous solution. We discuss how CHA is utilized on the solid sensor surface, including how the design of the FPL can generate sufficiently bright fluorescent spots. This concept is then applied in a sandwich immunoassay with interleukin 6 (IL6) as a model analyte.

## 2 Result and Discussion

**Figure 1:**
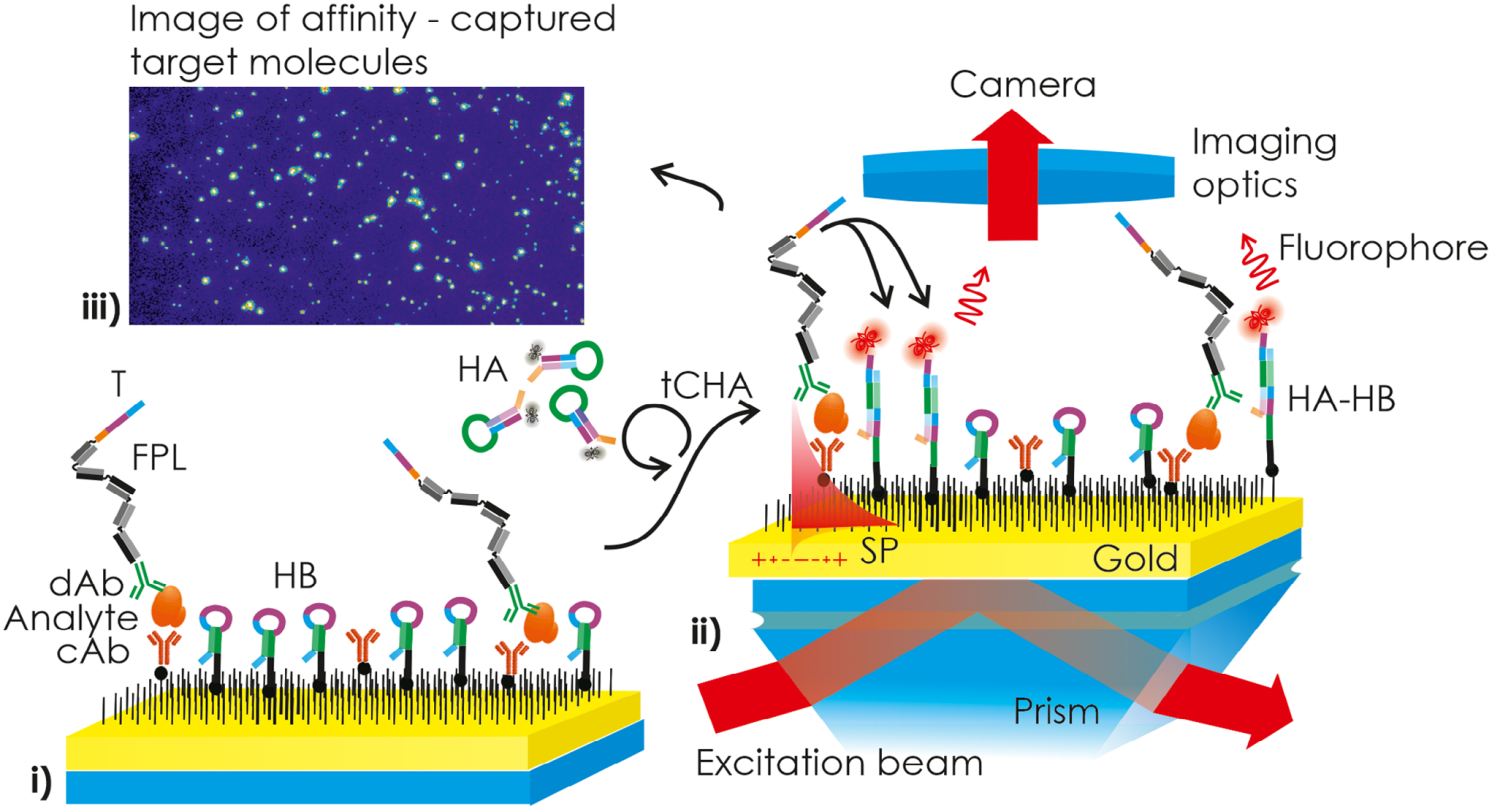
Schematic of the sandwich immunoassay with PEF readout of fluorescent spots generated by tCHA at locations where individual target analyte molecules were affinity captured.

The concept of the enzyme and partition‐free digital assay is shown schematically in **Figure** **1**. It relies on counting individual affinity‐captured biomolecules in a sandwich immunoassay format, enabled by PEF readout in combination with tCHA. The interface of the sensor is modified with a capture antibody (cAb, target analyte specific) and by additional DNA hairpin molecules (HB). After the affinity capture of target analyte by cAb, a sandwich is formed by its reaction with a detection antibody (dAb) that is conjugated with an FPL. This linker has at its outer end a trigger ssDNA strand (T) that is designed to react with another DNA hairpin molecule conjugated with a fluorophore (HA‐Cy5). HA‐Cy5 is dissolved in an aqueous solution that is contacted with the solid sensor surface after the formation of the sandwich. The sequences of HB and HA hairpins are selected to not directly react with each other in their closed form. However, their hybridization is made possible in a cyclic CHA manner in the presence of the trigger sequence T. Owing to the spatial localization of the reaction at positions where target analyte is captured, these events can be visualized as bright fluorescent spots with a size controlled by the length and flexibility of the FPL.

Sequences of the DNA strands used for tCHA are specified in the Supporting Information (Table S1, Supporting Information). Both HA‐Cy5 and HB hairpins were introduced in their closed form (see predicted structure in Figure S1, Supporting Information included in the Supporting Information and in prior investigations^[^ ^31^ ^]^). These hairpins were designed with an overhanging short ssDNA sequence acting as a toehold. The toehold of the closed HA‐Cy5 facilitates the interaction with the T sequence that allows for hybridization of the T‐HA duplex through TMSD.^[^ ^32^ ^]^ The conformation of HA‐Cy5 is then toggled from a closed to an open state. The open HA‐Cy5 molecule exposes a ssDNA sequence having an affinity for another toehold of the hairpin HB for their hybridization via TMSD. This second step is accompanied with the release of T strand from the intermediate T‐HA‐HB complex resulting in the formation of HA‐HB duplex on the sensor surface and liberation of T for the next reaction cycle. In the further discussed experiments, a gold sensor surface was modified with a mixed self‐assembled monolayer (SAM) composed of thiol molecules carrying both biotin and oligoethylene glycol (OEG) headgroups. OEG groups were used to suppress non‐specific interaction of biomolecules with the sensor surface, and biotin moieties were employed for the immobilization of nucleic acid and protein molecules conjugated with a biotin tag via neutravidin (NA).

As indicated in Figure 1, the measured fluorescence intensity *F* associated with the binding of HA‐Cy5 on the sensor surface was optically amplified by their coupling with surface plasmons. In this PEF method,^[^ ^28^, ^30^, ^33^ ^]^ the surface plasmon field (with exponentially decreasing electromagnetic field intensity) is resonantly excited at a wavelength of *λ*_ex_ = 632.8 nm that is coincident with the absorption band of the fluorescent Cy5 labels. Their confined field profile stretches to a short sub‐wavelength distance of ≈100 nm from the sensor surface, which leads to its increased strength in the near‐field compared to the optical excitation beam traveling in the far‐field. The accumulation of field intensity on the surface allows for facile measuring of fluorescence signal kinetics *F*(*t*) that is associated with surface reactions, and it is not masked by the background emission of molecules present in the bulk solution. In addition, the plasmonically increased strength of excitation field leads to enhanced fluorescence intensity by a factor of ≈45 specific for this setup (i.e., Kretschmann configuration and excitation wavelength).^[^ ^28^, ^34^ ^]^

As discussed further, the condition for cyclic CHA reaction on the sensor surface was first established by the help of PEF readout with fluorescence intensity *F* averaged over ensembles of molecules. Afterward, the PEF imaging setup was used to test a tCHA configuration for SMD with particular focus on a design of the FPL (i.e., how the FPL length affects the reaction speed). Finally, the implementation of tCHA with the PEF imaging is carried out for sandwich immunoassays specific to interleukin 6 (IL6) protein serving as a model target analyte.

### 2.1 CHA Cyclic Interaction on the Sensor Surface

**Figure 2:**
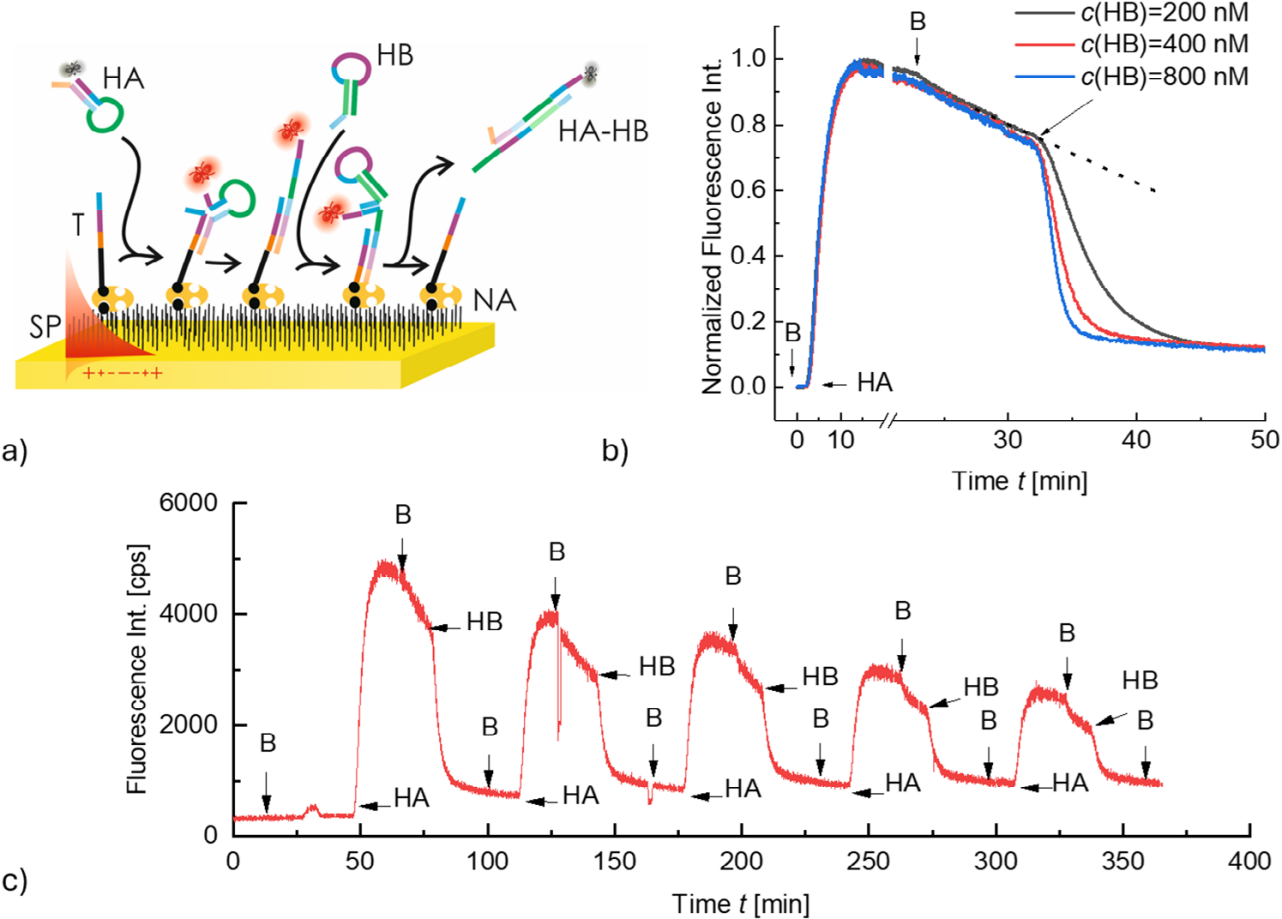
a) Schematics of the tCHA reaction with surface‐attached T sequence [immobilized via a biotin tag to neutravidin NA from a solution with *c*(T) = 10 nm]. b) PEF kinetics showing the affinity capture of HA‐Cy5 hairpin from a solution with *c*(HA‐Cy5) = 100 nm and its detachment via reacting with HB dissolved in a solution with *c*(HB) = 200, 400, and 800 nm. c) Example of the reversible sequential reaction of T with HA‐Cy5 and HB. All reaction steps are separated by rinsing with a working buffer denoted as B (the sharp decrease in fluorescence intensity at time *t* = 130 min is attributed to the introduction of air bubbles during the rinsing step, leading to detuning of SPR and decreasing of fluorescence signal).

Given the reliance of CHA on the cycling TMSD mechanism, we first tested the reversibility of the interaction between T, HA‐Cy5, and HB when these constituents are anchored to the solid sensor surface. Therefore, the gold sensor surface with attached NA molecules was first modified by a T strand conjugated to a biotin tag which, as schematically shown in **Figure** **2**a, was consecutively reacted with fluorescent HA‐Cy5 and by non‐fluorescent HB.

As shown in Figure 2b, the affinity binding of HA‐Cy5 to the sequence T attached to the surface is accompanied by a gradual increase in the fluorescence intensity *F* that saturates after 10 min. After a subsequent rinsing step with working buffer B, the measured fluorescence intensity *F* slowly decreases due to the gradual bleaching of the Cy5 emitters at the sensor surface. After 30 mins, the surface was reacted with non‐fluorescent HB hairpin, where the faster decrease of the fluorescence intensity *F* can be attributed to TMSD. The displacement speed can be controlled by the HB concentration, where for *c*(HB) = 200, 400, and 800 nm, the fluorescence signal *F* decreases with a rate that is 29, 64, and 93‐fold faster than the bleaching, respectively. The fluorescence intensity changes have an exponential character and it decreases to an intensity that is slightly higher than the original baseline (prior to the binding of HA‐Cy5 to surface‐immobilized T). Even with the finite recovery rate [of ≈85% for all tested concentrations *c*(HB)], the displacement of HA‐Cy5 by reacting with HB liberates the sequence T and allows for repeated reaction cycles as documented by Figure 2c.

The magnitude of the fluorescence intensity change occurring upon the affinity binding of HA‐Cy5 decreases with the number of cycles, which is in accordance with reduced recovery yield and possible gradual blocking of T molecules available for the cyclic CHA. It is worth noting that this yield is comparable to other DNA amplification technologies based on DNA strand displacement.^[^ ^35^, ^36^ ^]^ The incomplete dissociation could be caused by the unspecific binding of HA‐Cy5 to the poly(T) sequence, or other mechanisms such as π‐π stacking, ionic binding,^[^ ^37^ ^]^ or re‐hybridization of HA‐Cy5 or HA‐HB complex to T via the toehold region.^[^ ^38^ ^]^ The measured data herein are in line with previous CHA experiments reported for the bulk solution where all DNA components are present at the same time, and it proves the high sensitivity of TMSD‐based detection assays not only for conventional CHA,^[^ ^27^ ^]^ but also for both localized CHA^[^ ^23^ ^]^ and a DNA walker configuration^[^ ^39^ ^]^ when the amplification process occurs within a prepared circuit track such as DNA nanowire, and DNA‐decorated nanoparticle.

### 2.2 Design of Flexible Polymer Linker

**Figure 3:**
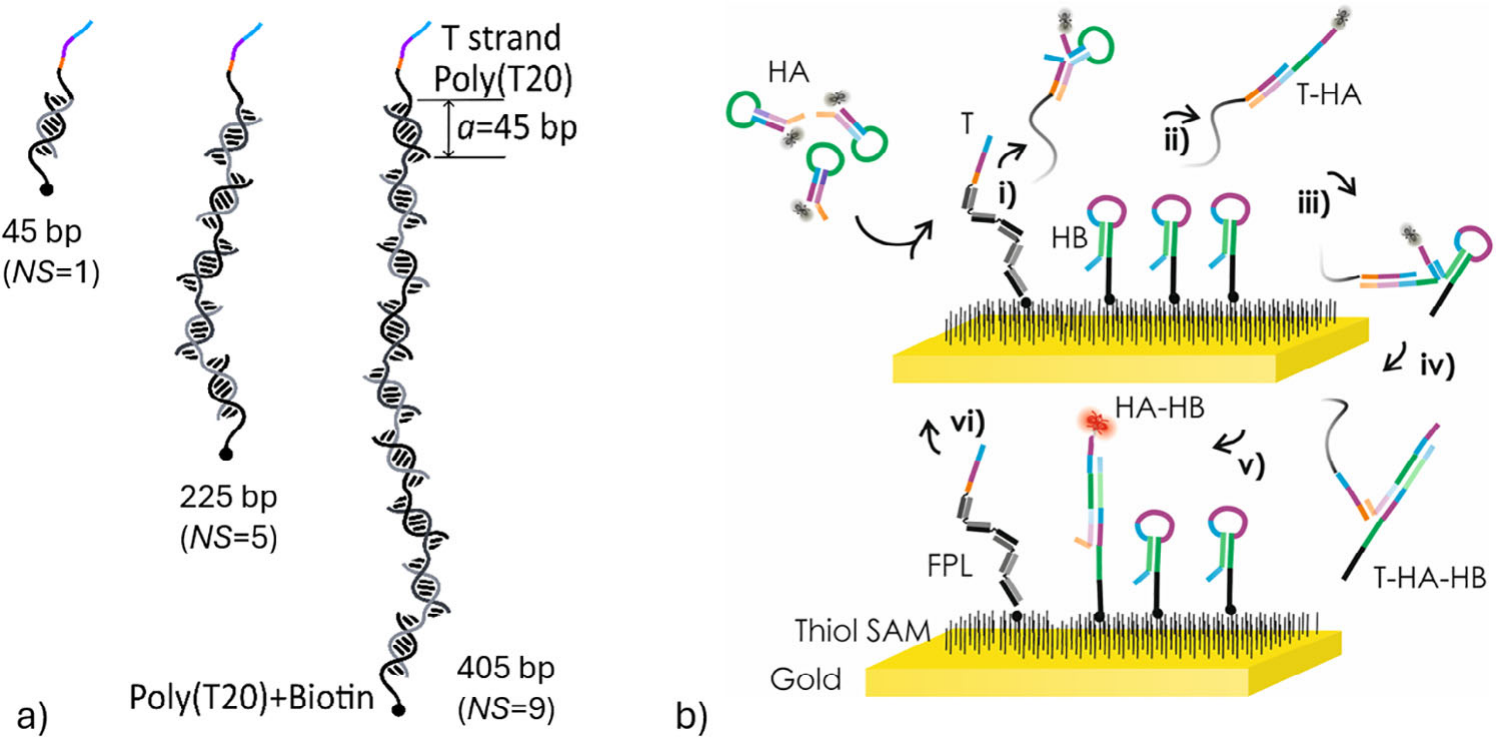
a) Design of an FPL composed of *NS* segments of dsDNA connected by ssDNA hinges. b) Schematic of a biointerface configuration for tCHA carrying HB sequences that are co‐immobilized with T coupled to an FPL for spatial confining of CHA cyclic reaction.

We tested FPLs with varied lengths composed of individual dsDNA segments of 45 bases, which can be assumed to behave as rigid rods with a length of *a* = 15.3 nm (when taking into account the dsDNA length of 0.34 nm per bp^[^ ^40^ ^]^ and a persistence length of ≈50 nm^[^ ^41^, ^42^ ^]^). As seen in **Figure** **3**a, we used three FPLs with a number of rigid segments set to *NS* = 1, 5, and 9 that were connected by non‐ligated ssDNA acting as flexible hinges due to the short ssDNA persistence length of <1 nm.^[^ ^43^, ^44^ ^]^ These FPLs were prepared from a pool of ssDNA strands by a thermal annealing method (as summarized in Figure S2, Supporting Information with the ssDNA strands specification provided in Table S2, Supporting Information). All FPLs were conjugated with sequence T on one end for triggering CHA, with a biotin tag on the opposite end serving for attachment to the metallic surface. Pre‐assembled FPLs were mixed with HB‐biotin molecules at a concentration ratio of 1:10^5^ [*c*(FPL‐T) = 10 fm and HB‐biotin at *c*(HB) = 1 nm] and reacted with the NA surface.

As shown in Figure 3b, we tested tCHA in a format such that HA‐Cy5 hairpins present in a solution were brought in contact with the sensor surface carrying co‐immobilized trigger T and hairpin HB sequences. The closed hairpin HA‐Cy5 does not directly react with the closed hairpin HB on the surface, however, this can occur after TMSD opening of HA‐Cy5 by the trigger sequence T that is initiated by its toehold (i). Then, the T‐HA duplex (ii) presents an overhanging ssDNA part that reacts with HB toehold (iii) and subsequently leads to the formation of tethered HA‐HB duplex (iv). Finally, the sequence T dissociates from T‐HA‐HB complex (v), which makes it available for the next reaction cycle (vi).

**Figure 4:**
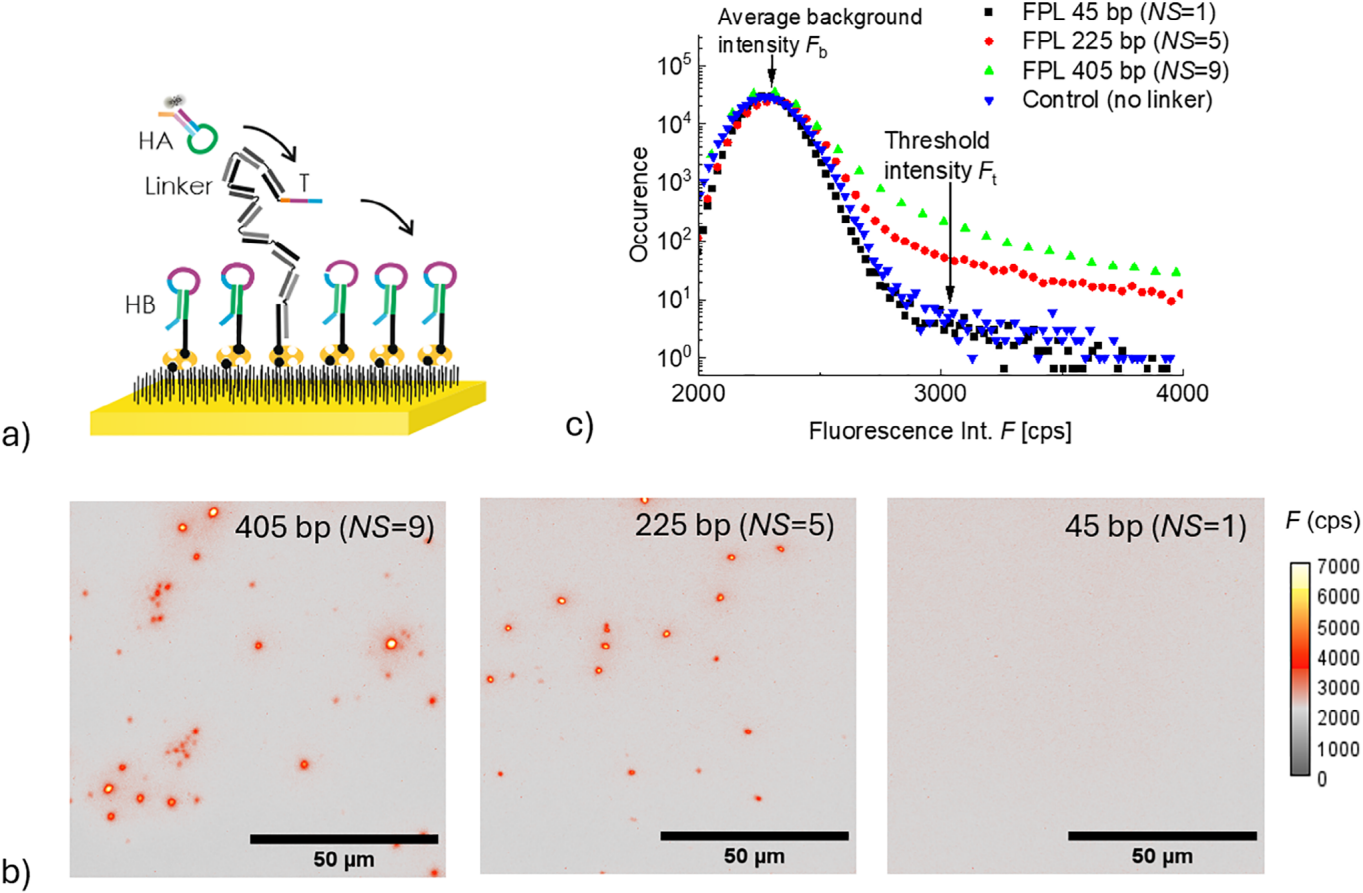
a) Schematic of the biointerface with FPL length of *NS* = 9, b) comparison of PEF images obtained for tCHA with three FPLs exhibiting lengths of 405, 225, and 45 bp, and c) respective histograms compared to the control. The biointerface was prepared by reacting the NA‐coated surface with a solution of *c*(T) = 10 fm and *c*(HB) = 1 nm, *c*(HA‐Cy5) = 10 nm and reaction time was 120 min.

This reaction is spatially localized with the use of FPL as schematically illustrated in **Figure** **4**a. Such confinement of CHA on the sensor surface is implemented to visualize individual reaction spots by PEF imaging, see Figure 4b. This imaging was performed after the tCHA reaction when the sensor surface was rinsed with a buffer and PEF images of an area 100 × 100 µm were captured. Histograms of measured fluorescence intensity (from individually acquired images) can be seen in Figure 4c. They exhibit a peak at ≈2400 cps, which corresponds to the average background signal *F*_b_ ascribed to both the leaking of excitation light through optical filters and the dark current of the CCD camera. At higher intensities, the histograms show a shoulder, where the occurrence values increase when prolonging the FPL length, which is a signature of individual fluorescent spots. At the onset of these shoulders, we defined a threshold intensity *F*_t_ for the identification of spot areas in the measured images.

Images in Figure 4b show that distinct fluorescent spots become apparent after tCHA for FPLs with 225 and 405 bp (*NS* = 5 and 9, respectively), while for the shortest FPL with 45 bp (*NS* = 1), the image did not qualitatively differ from the control (HB‐biotin without FPL immobilized on the surface). A quantitative analysis was carried out to count the number of fluorescent spots *NSF* (by using a threshold set to *F*_t_ = 3000 cps based on the determined histograms in Figure 4c) and the average spot intensity. For the FPL linkers with 405 bp (*NS* = 9) and 225 bp (*NS* = 5), a number of fluorescent spots of *NFS* = 38 and 19, respectively, was obtained. The fact that the shortest FPL with 45 bp (only one segment *NS* = 1) does not show evidence of tCHA can be attributed to its limited flexibility, hindering the ability to approach the tip with the trigger sequence T to the sensor surface with HB molecules. The use of FPLs with multiple rigid segments was observed to overcome this limitation and facilitate tCHA, which is manifested as measurable fluorescent spots. For *NS* = 9 segments the number of determined fluorescent spots was higher than for *NS* = 5 segments, which can be ascribed to the larger area that is reachable by each anchored FPL. The respective higher amounts of available HA‐Cy5 molecules around the FPL tethering point translate to a reaction with a higher probability to overpass the threshold.

### 2.3 Density and Brightness of Individual Fluorescent Spots

Let us note that the number of detected fluorescent spots *NFS* observed in the previous section on a footprint of 100 × 100 µm is substantially lower than the value obtained from FPL surface density (that can be estimated from the molar concentration ratio 1:10^5^ of FPL and HB in the aqueous solution reacted with the sensor surface). The measured surface mass density of immobilized NA was 4.14 ng mm^−2^ (see Figure S3, Supporting Information), which is close to a fully packed monolayer when taking into account the molecular weight of NA molecule as 60 kDa,^[^ ^45^ ^]^ and translates to an average distance between the points that can bind biotin‐tagged FPL or HB of ≈4.9 nm (see Section S2 in Supporting Information). Assuming all NA binding pockets were occupied and their probability of occupancy with FPL or HB scales with the respective concentration ratio, the maximum *NFS* would be ≈4.1 × 10^3^ on the PEF detection footprint, which is above the experimentally measured values by a factor of ≈10^2^. A more accurate non‐equilibrium analysis that takes into account diffusion‐limited transport and depletion along the flow channels reveals that the estimated number of captured molecules within the PEF imaging area is 96 for FPL‐T (10 fm) and 4.2 × 10^7^ for HB (1 nm) during a 30‐minute assay (Figure S6a, Supporting Information).^[^ ^46^ ^]^ These capture rates, derived from an analytical method detailed in the Supporting Information (Table S3, Supporting Information), further demonstrate that diffusion limitations and differences in diffusivity significantly influence molecule capture, with HB capture rates being ≈2.6 × 10^5^ higher than those of FPL‐T. However, these values are ≈10 × less than the total estimated number of binding sites, hence the diffusion‐limited approach can be considered to be free from any limiting effects due to equilibrium. Consequently, the non‐equilibrium analysis reveals that the experimental conditions used herein would lead to an NFS of 96, much closer to the experimentally measured value of 38 (Figure S6b, Supporting Information) fitting within a moderate factor of 3. Discrepancies between experiment and diffusion‐limited estimations can be attributed to a fraction of FPL with T that are not active due to not fully recoverable hybridization with HA and HB hairpins (see Figure 2c).

The overall brightness of each spot is expected to scale with the area around each FPL anchor on which fluorescent HA‐Cy5 can be docked. This area can be determined from the end‐to‐end distance *r* of FPLs. As illustrated in Figure 3a, the FPLs can be approximated as a string of rigid rods (length of ≈*a* = 15 nm) connected with flexible hinges. The end‐to‐end distance of such object *r* can be described based on 3D random walk theory^[^ ^47^, ^48^ ^]^ as the probability function *P*(*r*) (see the mathematical model in Equation S2 (Supporting Information) and the simulated end‐to‐end distance for FPLs in Figure S4 (Supporting Information) This model predicts the full width of half maximum of the probability function *P*(*r*) of 32 nm for the FPL with 225 bp (*NS* = 5) and of 42 nm for FPL with 405 bp (*NS* = 9). The end‐to‐end distances at the maximum probability are predicted as *r* = 27 and 37 nm for these configurations, respectively. These values are equivalent to the radius of gyration,^[^ ^49^ ^]^ and the area of a circle defined by these parameters is then obtained as 2.3 × 10^3^ nm^2^ (FPL 225 bp, *NS* = 5) and 4.3 × 10^3^ nm^2^ (FPL 405 bp, *NS* = 9). These areas can accommodate ≈0.95 × 10^2^ and 1.78 × 10^2^ NA molecules, respectively, that can be reacted with closed HB molecules when assuming the average area occupied by single NA is of 24.1 nm^2^.^[^ ^50^, ^51^ ^]^ These values represent the upper limit of the number of fluorophore emitters measured per individual bright spot (with a size below diffraction limit). Let us note that these numbers of emitters are similar to those reported for other approaches such as immuno‐RCA,^[^ ^52^ ^]^ and the degree of localization is comparable to other studies using related polymer systems.^[^ ^53^ ^]^

### 2.4 Kinetic Measurement of tCHA at Individual Spots

**Figure 5:**
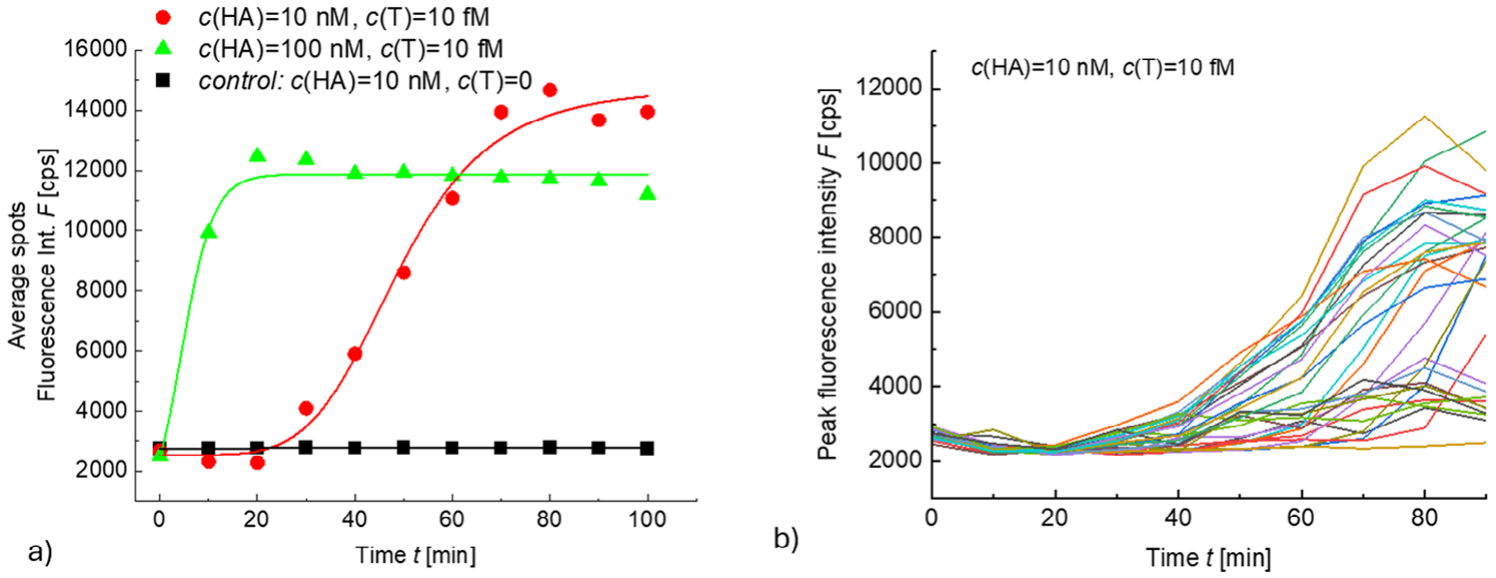
Time dependence of tCHA. a) Kinetics of the averaged fluorescence intensity of fluorescent spots associated to the affinity capture sequences T from a solution with *c*(T) = 10 fm via a linker, and b) comparison of respective signal *F* for individual spots in an experiment with *c*(HA) = 10 nm, *c*(T) = 10 fm.

tCHA was monitored in real time by using PEF imaging to investigate the speed of this developed protocol. Figure S5 (Supporting Information) shows an example of PEF images captured before and after tCHA was carried out on a biointerface with 405 bp FPL (*NS* = 9) that was conjugated with trigger T at its outer free end. Such images were acquired as a time series during the tCHA reaction, where the maximum fluorescence intensity was determined from localized spots. The progression of the maximum spot fluorescence intensity (average over the set of all identified spots) is compared for concentrations *c*(HA‐Cy5) = 10 and 100 nm, see **Figure** **5**a. For the lower concentration of *c*(HA‐Cy5) = 10 nm, the reaction reached saturation after ≈60 min, when all available HA binding sites in the vicinity to each FPL anchor are occupied with HA‐Cy5. When the concentration is increased to *c*(HA‐Cy5) = 100 nm, the reaction proceeds faster and the fluorescence intensity rises to the saturation value in less than 10 min. Importantly, the saturation intensity for both *c*(HA‐Cy5) yields similar values, where no fluorescent spots were observed in the control experiment (with immobilized *T* sequence to FPL).

The tCHA kinetics presented in Figure 5a were acquired by averaging values taken from individual spots and the outcome curves were fitted with a logistic function. In addition, we examined the spot‐to‐spot differences in the measured fluorescence signal associated with tCHA. As Figure 5b shows, the kinetics then exhibit rather spread equilibrium intensity with a non‐linear dependence and apparent decrease in the peak intensity *F* at longer times. The possible reason for this behavior can be the gradual bleaching and competing interaction routes that can be ascribed to the finite tCHA recovery yield. Despite this observed heterogeneity, these results confirm that tCHA translates the presence of T sequence to measurable fluorescent spots, and the trends agree with the prior results presented in Figure 2.

### 2.5 Model Assay for Detection of Nucleic Acid Analyte

**Figure 6:**
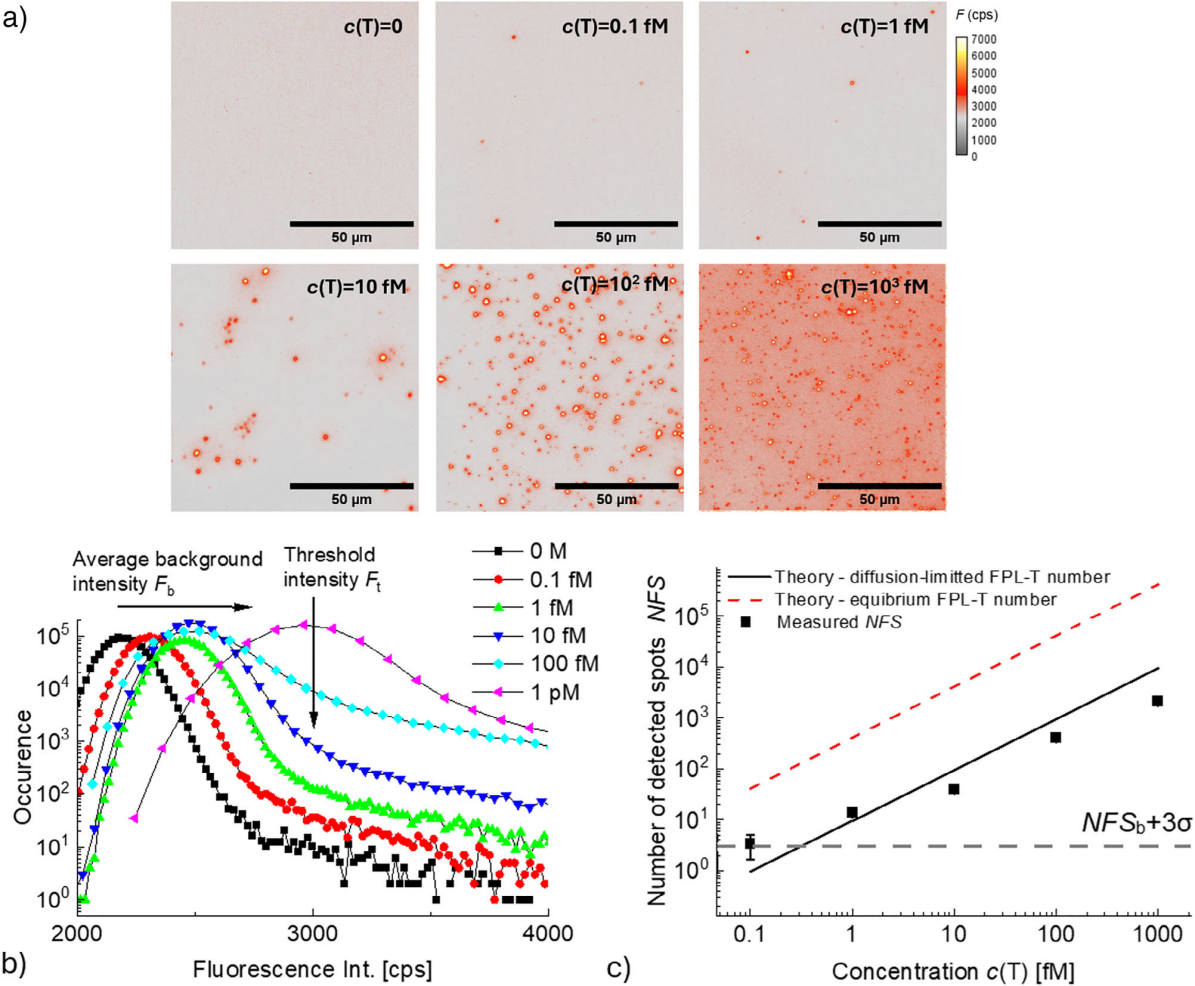
a) Series of acquired PEF images after tCHA for the concentration *c*(T) of 0.1, 1,10, 10^2^, and 10^3^ fm compared to the control experiment *c*(T) = 0. tCHA reaction was implemented with a length of DNA linker of 405 bp (*NS* = 9), *c*(HB) = 1 nm, *c*(HA) = 10 nm, and reaction time of *t* = 120 min. b) Histograms of fluorescence intensity for acquired images of tCHA at variable concentration *c*(T). c) dependence of the sensor output (number of fluorescent spots *NFS*) on *c*(T), with a minimum detectable concentration compared to a diffusion‐limited transfer model. The data points are the average values from three different observations with error bars representing mean ± SD.

To implement tCHA into assays with single molecule discrimination, this assay must be utilized so the presence of T sequence can be associated with the affinity capture of target analyte on the sensor surface. Thus, we tested the ability to determine individual binding spots as a function of concentration of T sequence present in aqueous solution reacted with the sensor surface *c*(T). As shown in **Figure** **6**a, the density of fluorescent spots observed on a footprint of 100 × 100 µm clearly increases with the concentration *c*(T). The intensity profiles of all images are presented in Figure 6b as histograms, where a threshold of fluorescence intensity *F*_t_ was set to 3000 counts to distinguish the spots against the background. It should be noted that for concentrations *c*(T)>1 pm, the ability to distinguish individual spots becomes limited as their increased density leads to spot overlapping. This is manifested as increased background and a respective shift and broadening of the background peak *F*_b_ present in the histogram plots in Figure 6b. For tCHA with FPL *c*(T) = 1 pm, the background fluorescence peak *F*_p_ shifts to a value that is above the used threshold *F*_t._ and thus compromises the ability of counting fluorescent spots.

Figure 6c shows the number of fluorescent spots *NFS* plotted against the concentration *c*(T). It shows that *NFS* is proportional to the concentration *c*(T) in the range from 0.1 fm to 1 pm; for the blank solution with *c*(T) = 0, the average background false positive number of sensing spots was determined as *NFS_b_* = 1, with three times standard deviation of 3σ = 3. The measured *NFS* values match very well to estimates of diffusion‐limited capture (represented as a black line) and falling within a factor of 3× from the diffusion‐limited estimate, which further confirms that under these conditions, both HB and FPL‐T do not occupy all the NA binding sites. The relationship between experimental data and diffusion‐limited estimation changed at the lower concentration of FPL‐T < 1 fm, with higher‐than‐expected experimental values. This might be due to the nonspecific generation of fluorescence spots, which become more pronounced at such low concentrations (in addition to larger standard deviations). The detection limit (LOD) of the implemented tCHA for T sequence was then calculated as 0.12 fm, where the fitted *NFS* function intersects the value of *NFS*_b_+3σ. This experimental LOD also fell within a factor of 3× from the diffusion‐limited estimate, which supports the agreement between the experimental and theoretical results (summarized in more detail in Figure S6b, Supporting Information). The detection range for this single molecule readout has an upper concentration range of ≈1 pm, above which the high density of individual spots leads to their strong overlapping. However, above this upper concentration limit, the readout can be evaluated in a standard regime relying on averaging over ensemble of captured molecules based on determining the mean fluorescence intensity.^[^ ^52^ ^]^

### 2.6 Sandwich Immunoassay with tCHA

**Figure 7:**
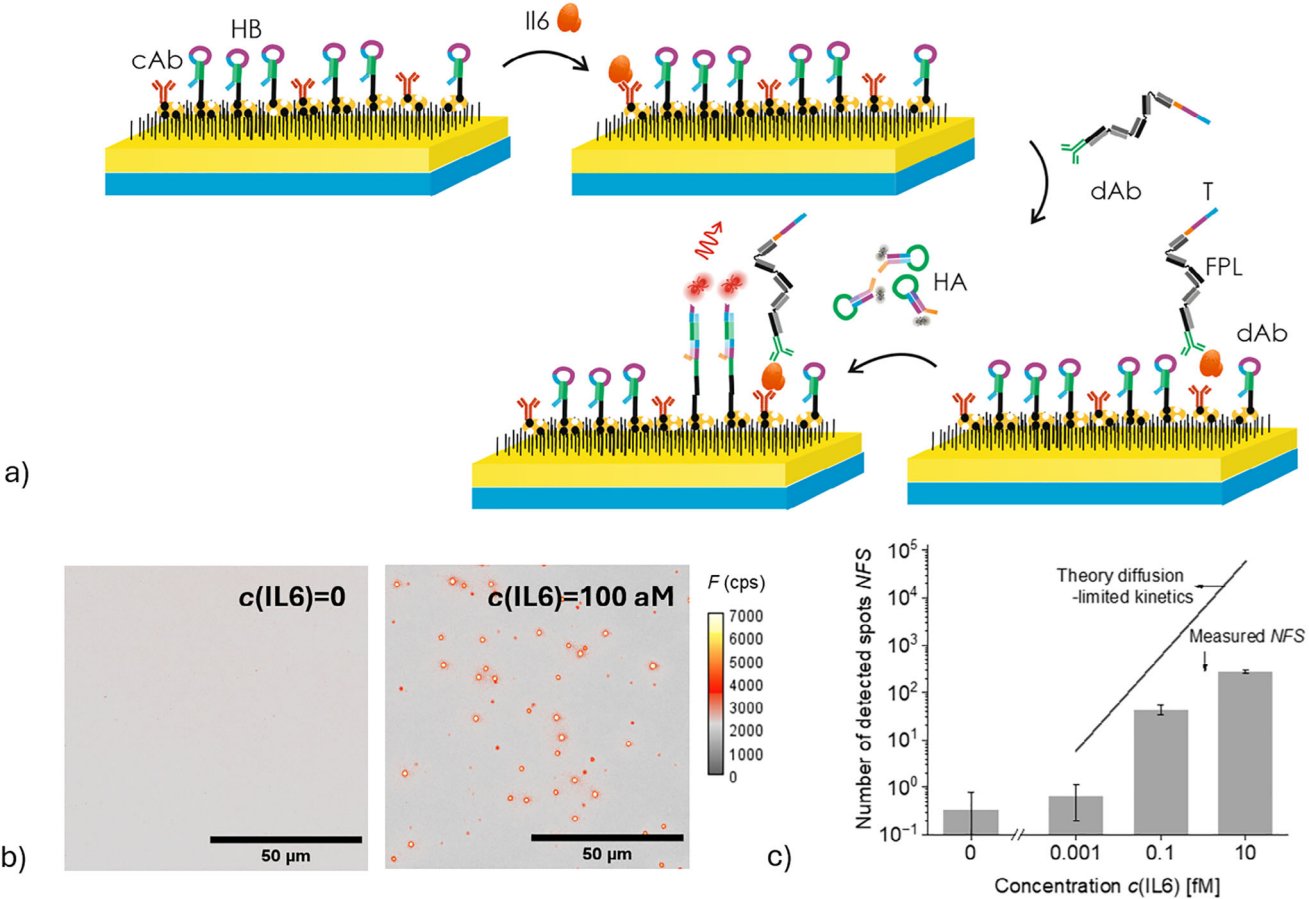
a) Schematic of the sandwich IL6 assay with tCHA amplification with b) comparison of the fluorescence images acquired for the control sample with *c*(IL6) = 0 and *c*(IL6) = 100 am (tCHA reaction time was set to 120 min. For this biointerface, cAb and HB molecules were immobilized on the neutravidin sensor surface by reaction with a solution with concentrations of *c*(cAb‐biotin) = 1 pm and *c*(HB‐biotin) = 1 nm). c) Comparison of the number of detected spots in a concentration range of *c*(IL6) = 0 to 10 fm. The detected *NFS* across the concentration *c*(IL6) = 1 am to 10 fm in comparison with the theoretical model of mass transfer. The data points are the average values from three different observations with error bars representing mean ± SD.

The approach of PEF imaging of individual tCHA fluorescent spots was finally tested using a sandwich immunoassay using a detection antibody dAb conjugated with a short oligonucleotide tag. Herein, we used maleimide‐thiol coupling and a short nucleotide incorporated onto a base FPL (with *NS = 9*) that was modified to carry a thiol group (instead of biotin used in the previous experiments). As illustrated in **Figure** **7**a, a tCHA‐based sandwich immunoassay was tested by using a plasmonic gold sensor surface modified with a mixed thiol SAM carrying biotin groups that were employed for the formation of NA monolayer, followed by the immobilization of biotinylated cAb and HB‐biotin. The biotinylated cAb molecules, which were specific to the chosen target analyte IL6, were mixed with HB‐biotin strands in aqueous solution at a concentration ratio 1:10^3^ [*c*(cAb‐biotin) = 1 pm and HB‐biotin at *c*(HB‐biotin) = 1 nm] and reacted with thiol SAM in the immobilization step. This concentration ratio was derived from the results above to yield maximum density of spots that are possible to distinguish without hindering caused by their spatial overlapping. In addition, one can expect that the chosen ratio *c*(cAb‐biotin)/*c*(HB‐biotin) provides >10^2^ binding sites per FPL and will avoid competing between neighboring tCHA reactions. The yielded biointerface thus offers affinity‐capturing of the target analyte molecules and furthermore, allows us to translate these binding events to bright detectable fluorescent spots upon the tCHA amplification phase.

The IL6 assay consisted of incubating the sensor surface with a sample solution spiked with IL6 molecules [at a concentration *c*(IL6) = 1 am, 100 am, and 10 fm] followed by their affinity binding to dAb conjugated with FPL [*c*(dAb) = 1 µg mL^−1^] in order to form a sandwich. In the subsequent amplification phase, HA‐Cy5 dissolved at a concentration of *c*(HA‐Cy5) = 10 nm was reacted with FPLs and neighboring HB molecules in cyclic manner based on the previously optimized tCHA mechanism. Figure 7b shows an example of measured PEF image obtained after tCHA was carried out for *c*(IL6) = 100 am as well as a control experiment with *c*(IL6) = 0 (additional experiments without assay constituents are included in the Supporting Information – Figure S7, Supporting Information). These images clearly show that the presence of IL6 molecules in the sample gives rise to *NFS* = 44 spots, while in the control experiments, the *NFS* was less than 1. In the examined concentration range of *c*(IL6) from 1 am to 10 fm, we calculated *NFS* following the same image analysis protocol, the results of which are plotted against the concentration *c*(IL6) as presented in Figure 7c. It shows that *NFS* increases with *c*(IL6) and when plotted in the double logarithmic scale, the fitted linear function has a slope close to 0.60 and exhibits a correlation coefficient of 0.97. The value of the linear slope is consistent with prior results obtained from the model assay that the T sequence served as the target analyte as presented in Figure 6.

The value of *NFS* of our assay was found to be 0.34% of the total number of IL6 molecules present in the solution that was analyzed. This level is consistent with other affinity‐capture‐based SMD methods^[^ ^54^, ^55^, ^56^ ^]^ as well as partitioning‐based digital bioassay (see Table S4, Supporting Information for comparison of benchmarks), where the number of detected target molecules is inherently limited by several factors such as the diffusion‐limited mass transfer of analyte to the surface, finite association and dissociation rates of used biomolecules, and the density of biorecognition elements on biointerface. Despite these effects, PEF‐tCHA approach was successfully adapted for detecting individual IL6 molecules present in the analyzed sample below femtomolar concentration.

## 3 Conclusion

This work reports on a new concept for digital readout of bioassays that do not require partitioning the analyzed sample together with enzymatic reactions. It relies on dual amplification strategy that is based on plasmon‐enhanced fluorescence – PEF – and tethered catalytic hairpin assembly – tCHA. This approach was carried out using a novel biointerface featuring a flexible polymer linker – FPL – that was self‐assembled from ssDNA strands to spatially confine the catalytic hairpin assembly reaction and associate the presence of affinity‐captured target molecules on the sensor surface with spatially distinguishable fluorescent spots. The brightness of these spots can be controlled by the design of the FPL, where we examined the effect of its length and flexibility based on a series of experiments that were carried out with the use of PEF imaging. The performed study reveals that tCHA allows to generate bright fluorescent spots (with up to ≈10^2^ emitters), at speeds that enable shortening the amplification to less than 10 minutes, where the monitoring of reaction kinetics at individual spot levels is shown to offer a means to investigate heterogeneities in the reaction. The reported implementation shows that for both ssDNA detection and a sandwich immunoassay, a sub‐fm limit of detection with detection range spanning up to 1 pm can be achieved when the area of 10^4^ µm^2^ is optically probed. This single molecule detection – SMD – concentration window can provide facile extension of conventional ensemble‐averaged detection range, which typically cannot access < pm concentrations. In terms of limit of detection, detection range, and analysis time the achieved performance positions this reported tCHA‐PEF assay at the forefront among other reported digital assays (LOD in 10^−1^–10^2^ fm concentration range, upper detection range at concentrations 1 pm – 10 nm, and detection time from 15 min to 5 h as summarized in Table S4 (Supporting Information), which focuses mostly on approaches relying on non‐enzymatic amplification without partitioning of the analyzed sample).

The overall number of detected spots indicates that the limit of detection can be improved by about two orders of magnitude by addressing mass transfer‐limited reaction kinetics and other effects that decrease detection yield of target molecules (<1% of target molecules present in the sample are detected in form of a bright fluorescent spot on the sensor chip surface). Further improvements in the optical readout – probing a larger sensor chip area without the need of high‐end fluorescence camera – are expected to be possible by capitalizing on the optical PEF amplification for future ultrasensitive and multiplexed digital readout assays. For instance, stronger confined electromagnetic field by plasmonic nanostructures is expected to increase the excitation rate and spatial resolution of fluorescence imaging, which will further improve the LOD along with a wider detection range.^[^ ^57^ ^]^ Moreover, robustness and specificity when analyzing complex biological fluids are of utmost importance for practical implementation in diagnostics. In future research, this performance will be pursued with the use of antifouling polymer coatings currently explored in other versions of enzyme‐based SMD in our laboratory.^[^ ^52^ ^]^ By addressing these factors together with long‐term stability, tCHA‐PEF holds the potential to serve as a foundation for advanced ultrasensitive diagnostics in clinical and point‐of‐care applications capitalizing on its partition‐ and enzyme‐free character.

## 4 Experimental Section

### Materials

The oligo (ethylene glycol) (OEG)–thiols [OEG–OH, HS–(CH_2_)_11_–EG_6_–OH, prod. no. TH 001‐m11.n6; OEG–biotin, HS–(CH_2_)_11_–EG_6_–biotin, prod. no. TH 004‐m11.n6; OEG–COOH, HS–(CH_2_)_11_–EG_6_–OCH_2_–COOH, prod. no. TH 003‐m11.n6] were obtained from ProChimia Surfaces (Poland). Phosphate‐buffered saline (PBS, pH 7.4, cat. no. E504), nuclease‐free water (NFW, cat. no. E476), Tween 20 (cat. no. 437082Q), and 99.9% pure ethanol (cat. no. 1.11727) were from VWR (Austria). As a working buffer (PBST), PBS was used with 0.05% (v/v) Tween 20. Magnesium chloride hexahydrate (cat. no. 7791‐18‐6), sucrose (cat. no. S7903), Trizma‐hydrochloride solution (Tris‐HCl, pH 8.0, 1 m, cat. no. T2694), dimethylsulfoxide (DMSO, 99.9% pure, cat. no. 41640‐M), Ethylenediaminetetraacetic acid disodium salt dihydrate (EDTA, cat. no. 324 503), acetic acid (cat. no. W200611) were purchased from Sigma‐Aldrich (Germany). Tris‐EDTA (TE) buffer (10 mm Tris and 1 mm EDTA, cat. no. AM9860) was purchased from Thermo Fisher (US). BSA‐biotin was purchased from Thermo Fisher (US, cat. no. 29 130, Pierce Bovine Serum Albumin, Biotinylated). As a folding buffer of DNA hairpin and DNA linker, Tris‐Acetate‐EDTA buffer containing magnesium ion (TAE+Mg) was prepared at 10x concentration (125 mm MgCl_2_, 400 mm Tris, 200 mm acetic acid, 10 mm EDTA). Dithiothreitol (DTT, cat. no. R0862molecular biology grade) was purchased from Thermo Fisher (US) and dissolved as 10 mm DMSO solution. Sulfo‐SMCC (sulfosuccinimidyl 4‐(N‐maleimidomethyl)cyclohexane‐1‐carboxylate) (cat. no. A39268) was purchased from Thermo Fisher (US). Neutravidin (NA, cat. no. 31 050), and Zeba spin desalting columns (7k MWCO, 0.5 mL, cat. no. 89 882 and 40k MWCO 0.5 mL, cat. no A57759.). DNA sequences specified in Table S1 (Supporting Information) were obtained from Integrated DNA Technologies (Belgium). The purified monoclonal rat anti‐IL6 antibodies (clone MQ2‐13A5 with product number 14–7069 and MQ2‐39C3 with product number 14–7068) were purchased from Invitrogen (Austria) and eBioscience (Austria), while recombinant human IL6 was obtained from Abcam (Austria, product number ab198571).

### Sensor Chip Preparation

Substrates made of BK7 glass were cleaned for 1 h by the Piranha cleaning procedure (75% of sulfuric acid and 25% of hydrogen peroxide). Then, they were subsequently sonicated with ultrapure water (*R* ≥ 18.2 MΩ cm^−2^), Hellmanex III 1% (v v^−1^), and ethanol, rinsed with pure ethanol, and dried by a stream of pressured air. The cleaned substrates were loaded into a thermal evaporator Auto306 Lab Coater from HHV Ltd (UK), after which a 2.6 nm thick chromium layer and 50.55 nm thick gold layer from MaTeck (Germany) were deposited (see Figure S8, Supporting Information for further characterization of the deposited metals by fitting of the optical response with a Fresnel reflectivity model). The gold‐coated slides were incubated overnight in ethanolic solution with dissolved thiols bearing hydroxyl and biotin headgroups (1 mm, mixed at a molar ratio of 1:5). After the mixed thiol self‐assembled monolayer (SAM) was formed, the chips were rinsed with pure ethanol and stored under an Ar atmosphere in the dark.

### Preparation of FPL and DNA Hairpins

Hairpin DNA probes (HA, HB, and HB‐biotin) were dissolved at 1 µm concentration in TAE buffer. The solution was denatured at 95 °C for 5 min and cooled down to 4 °C with decrease rate of temperature −1 °C min^−1^. The probes were stored at 4 °C in TAE + Mg and were used for further experiment by diluting with PBST. Three DNA FPLs with different lengths (405, 225, and 45 bp in order for respective FPLs to maintain the number of segments *NS* = 9, 5, and 1) were assembled by the thermal annealing method from a pool of DNA strands (shown in Table S1, Supporting Information; Figure 3a). The prepared sample was used for experiment without removing excess DNA strands. The DNA FPL was stored at 4 °C in 1x TAE + Mg buffer and was used for further experiment by diluting with PBST.

### Sandwich Immunoassay

The IL6 antibody clone MQ2‐39C3 (0.5 µg mL^−1^ in PBS) with a biotin tag served as capture antibody – cAb. The IL6 antibody clone MQ2‐13A5 (0.5 µg mL^−1^ in PBS) was used as detection antibody – dAb – which was first reacted with a sulfo‐ SMCC ester. The ester was dissolved in DMSO at a molar concentration of 10 mm, added in 100 or 80 molar excesses to the aqueous solution with dAb, and incubated on a shaker for 1 hour at room temperature following the manufacturer's procedure. Excess molecules were removed with spin desalting columns (40K MWCO) after the reaction. The thiolated Strand5 sequence was prepared by dissolving in 1x TE buffer with DTT (10 mm) for reduction of the disulfide groups to a mono‐sulfide moiety. The solution was passed through a spin desalting column (7K MWCO) for the removal of DTT immediately before the addition of the DNA to the SMCC‐antibody solution in a molar excess of 25. The reaction was incubated on a shaker for 3 h at room temperature and stopped by the removal of excess molecules with a spin desalting column (40 K MWCO). The dAb conjugated with ssDNA Strand5 complex was stored at ‐20 °C until use.

### Signal Amplification Based on tCHA

For testing of reversibility of CHA reaction steps, the thiol biotin SAM–modified chips were reacted with NA (1.67 µm in PBST) for 10 min followed by the binding of T‐biotin (50 nm) strand for 5 min. HA‐Cy5 (100 nm) was incubated with the sensor surface for 20 min followed by rinsing with PBST for 20 min, reacting with HB at a concentration of 200, 400, and 800 nm for 30 min and rinsing with PBST again prior starting the next cycle.

For PEF imaging of tCHA with DNA FPL, the gold sensor surface having a thiol biotin‐SAM was modified with 3 layers of NA supported by two intermediate layers of biotin‐BSA in order to increase the distance of emitters from the surface. After preparing the 1st layer of NA on the surface from a solution with *c*(NA) = 1.67 µm flowed for 30 min, a solution with *c*(biotin‐BSA) = 100 nm was flowed for 10 min before the next cycle. Rinsing with PBST was performed for 10 min after each reaction step. DNA FPL and HB‐biotin were co‐immobilized onto the multi‐layered NA surface by flowing their mixture prepared with controlled molar ratio. Finally, tCHA reaction was initiated by contacting the sensor surface with HA‐Cy5 molecules dissolved in PBST and after chosen time the reaction was terminated by rinsing with PBST.

For PEF imaging of tCHA implemented in immunoassay form, the same multi‐layered NA was used as the binding surface for the co‐immobilization of HB‐biotin and cAb‐biotin dissolved in PBST (at concentrations of *c* = 1 nm and 1 pm, respectively) and flowed for 30 min over the surface (see Figure S9, Supporting Information, further characterization clarified the immobilization of each biomolecule). After rinsing with PBST, target analyte IL6 spiked into Tris buffer (10 mm Tris‐HCL, 50 mm NaCl, pH 8.4) was flowed for 30 min to bind with the cAb on the surface. The dAb+Strand5 in PBST was then flowed for 30 min to complete sandwich immunoassay by binding with IL6. After rinsing with PBST, a DNA FPL with 405 bp (*NS* = 9) was dissolved in PBST at concentration of 1 nm and flowed through the sensor for 30 min to hybridize with Strand5 conjugated with dAb. Finally, HA‐Cy5 at *c*(HA‐Cy5) = 10 nm in PBST was flowed for 120 min for the initiation of tCHA. The amplification was terminated by rinsing with PBST.

### SPR and PEF Measurements on Ensembles of Molecules

The optical instrument used herein relied on the Kretschmann configuration of attenuated total reflection (ATR) method. The sensor chip was optically matched to a 90° LASF9 glass prism by a refractive index immersion oil and a HeNe laser beam (*λ*_ex_  = 632.8 nm, power 5 mW) was made incident onto its surface under a resonant angle *θ*_SPR_ set by a rotation stage with a stepper motor. The spectrum of the *λ*_ex_ beam was cleaned by a band‐pass filter (FL632.8‐10 from Thorlabs) and the power was adjusted by neutral density filters. An excitation beam at a wavelength of *λ*_ex_ was transversally magnetically polarized with a polarizer (LPVIS100 from Thorlabs, Newton, NJ, USA) and after reflecting from the sensor surface its intensity was measured with a photodiode detector connected to a lock‐in amplifier (7260 from EG&G). Against the sensor surface with a thin gold film, a transparent flow cell was attached (flow chamber volume of 10 µL) and a peristaltic pump REGLO Digital MS‐4/8 from Ismatec (Switzerland) was used to transport liquid sample solutions with a flow rate of 10 µL min^−1^. This flow cell (forming a reaction chamber) was sealed to a silica glass substrate (with drilled inlet and outlet ports) by a polydimethylsiloxane gasket; fluidic connections were made with Tygon tubing with 0.25 mm inner diameter snug fit onto short tubing sections attached to each drilled inlet/outlet. By closing the loop with the tubing, the sample solutions were continuously reintroduced. Cy5 fluorophores at the sensor surface were excited by the enhanced intensity of SPs at *λ*_ex_, and the fluorescence light emitted perpendicular to the surface at a Stokes‐shifted wavelength of *λ*_em_ = 670 nm was collected through the transparent flow‐cell, collimated by a lens with a focal length of *f* = 30 mm (LB1471 from Thorlabs, UK), and made passing through a laser notch filter (LNF, Melles Griot, XNF‐632.8‐25.0 M CVI, USA) and two bandpass filters (FBP, Thorlabs, FB670‐10 and 670FS10‐25 from Andover Corporation Optical Filter, USA), and its intensity *F* was detected by a photomultiplier (H6240‐01, from Hamamatsu, Japan) connected to a counter (53131A, *f* = 225 MHz, Agilent Technologies, USA).

The reflected beam intensity *R* in % and the fluorescence intensity *F* in counts per second (cps) were recorded as a function of time *t* or the angle of incidence *θ* by using the software Wasplas (developed at Max Planck Institute for Polymer Research in Mainz, Germany). The angular reflectivity spectra were measured and the SPs excitation angle *θ*_SPR_ was determined by the angle where the SPR dip minimum occurs. Angular reflectivity spectra were measured, and to characterize the thickness and refractive index of the immobilized biomolecule, they were fitted by a Fresnel reflectivity‐based model in the software tool Winspall (developed at Max Planck Institute for Polymer Research in Mainz, Germany).

### PEF Imaging of Individual Molecules

For the imaging of tCHA‐generated fluorescent spots on the sensor surface, the ATR optical instrument with Kretschmann geometry was modified as follows. A sensor chip was assembled from a glass substrate coated with thin gold film and a microfluidic system with arrays of channels. The microfluidic component was composed of an adhesive gasket ( 7952MPL, 50 µm thick from 3M ) onto which channel designs were cut (Silhouette cameo 4) and sealed to a coverslip of 170 µm thickness (CG15KH1 from Thorlabs Inc. UK) to form reaction chambers. The input and output ports to the microchannels were cut by a PERLA 100 laser system into a glass coverslip and connected to Tygon with 0.13 mm inner diameter tubing with an acrylic resin. A peristaltic pump transported the liquid sample solutions through the flow microchannels with a cross‐section of 1000 × 50 µm (and a volume of <1 µL) at a flow rate of 10 µL min^−1^. The optical configuration used for PEF detection using photomultiplier was replaced by an electron multiplying charge‐coupled device (EMCCD, iXon 885K from Andor Technology, Belfast, UK) camera, a tube lens with 200 mm focus length (TTL200‐B from Thorlabs, UK), and a 40× objective lens (RMS40X from Olympus, Japan). The emitted fluorescence light was imaged by a 40× objective lens to infinity, passed through the laser notch filter (LNF, XNF‐632.8‐25.0M  CVI Melles Griot, USA) and the band pass filter (FBP, Thorlabs, FB670‐10 and 670FS10‐25 from Andover Corporation Optical Filter, USA) before traveling through a tube lens (TTL200 from Thorlabs Inc, UK) at being imaged at the EMCCD camera (operated at −70 °C and with EM gain set to 100). Fluorescence images were acquired in time series with a period of 10 min, accumulation of 6, and exposure time of 10 s. Acquired images were exported in 16‐bit TIFF format by Andor Solis software (Andor Technology, UK) and further processed in ImageJ (Rasband, W.S., US National Institutes of Health, USA). By applying a threshold *F*_t_, it was converted to a 1‐bit image, and the built‐in routine, Analyze Particles function, was used to classify clusters of pixels as fluorescence bright spots prior to their counting. By applying the built‐in distribution function in Origin 2023b software intensity of identified bright spots was determined.

### Statistical Analysis

All data, including error bars in figures, are expressed as mean ± SD. Sample sizes (*n*) for each analysis are provided in the figure legends. The fitting of measured curves was carried out by modules in Origin 2023b for non‐linear fitting analysis. All numerical analysis and figure plotting were performed using Microsoft Excel and Origin 2023b. All obtained PEF images exported from Andor Solis software are processed without pre‐processing as described above in the Experimental Section, using the built‐in function of ImageJ in order to determine fluorescence spots.

## Conflict of Interest

The authors declare no conflict of interest.

## Author Contributions

The manuscript was written through the contributions of all authors. All authors have approved the final version of the manuscript.

## Supporting information



Supporting Information

## Data Availability

The data that support the findings of this study are openly available in Zenodo at 10.5281/zenodo.14067375, reference number 0.
