# Supplementary material for: Tethered Catalytic Hairpin Assembly with Plasmon‐Enhanced Fluorescence Readout for Single Molecule Detection
Source: Small Methods. 2025 Apr 10;9(8):2500037. doi: 10.1002/smtd.202500037 (PMC12391651; doi:10.1002/smtd.202500037)
Supplement: Supplementary file 1 — Supporting Information [file SMTD-9-2500037-s001.pdf]

small
methods

Supporting Information

for *Small Methods*, DOI 10.1002/smtd.202500037

Tethered Catalytic Hairpin Assembly with Plasmon-Enhanced Fluorescence Readout for Single Molecule Detection

*Naoto Asai, Katharina Schmidt, Gizem Aktuğ, Stefan Fossati, Juraj Sladek, N. Scott Lynn Jr. and Jakub Dostalek**

Supporting Information

Tethered Catalytic Hairpin Assembly with Plasmon-Enhanced Fluorescence Readout for Single Molecule Detection

*Naoto Asai, Katharina Schmidt, Gizem Aktuğ, Stefan Fossati, Juraj Sladek, Nicholas Scott Lynn Jr., Jakub Dostalek**

N. Asai, K. Schmidt, J. Dostalek*

LiST – Laboratory for Life Sciences and Technology, Faculty of Medicine and Dentistry, Danube Private University (DPU), Steiner Landstraße 124, 3500 Krems-Stein, Austria

E-Mail: (jakub.dostalek@dp-uni.ac.at)

G. Aktuğ, S. Fossati, J. Sladek, N. S. Lynn, J. Dostalek*

FZU-Institute of Physics, Czech Academy of Sciences, Na Slovance 2, Prague 182 21, Czech Republic

G. Aktuğ

Faculty of Mathematics and Physics, Charles University, Prague 121 16, Czech Republic

Table of content:

1. Sequences of ssDNA oligos serving in tCHA and predicted structure of hairpin DNA

2. Assembly of a FPL composed of ssDNA oligos

3. Prediction of average distance between NA molecules

4. Prediction of the end-to-end distance of FPL

5. Time-series PEF images of tCHA

6. Analysis of diffusion-limited analyte binding

7. Control experiment for immunosandwich-tCHA assay

8. Comparison of benchmarks on SMD and digital assay

9. Characterization of the deposited metal layers

10. Additional characterization of cAb and HB immobilization

11. References

1. Sequences of ssDNA oligos serving in tCHA and predicted structure of hairpin DNA

The used sequences of ssDNA oligos in CHA reaction are presented in **Table S1** with color and underline - marked specific segments that carry complementary sequences. Italic - highlighted parts are overhanging sequences acting as a toehold region for hairpin DNAs in a closed loop structure, as predicted in **Figure S1**.

Table S1 Sequence of used DNA stands in tCHA.

Name	5′ → 3′
HA	*CCCAGTC*TTATAGGTAGGTTATCCCCATGTGTAGAGGATAACCTACCTATAA - Cy5
HB	*GGTTATC*CTCTACACATGGGGATAACCTACCTATAACCATGTGTAGAG
HB-Biotin	*GGTTATC*CTCTACACATGGGGATAACCTACCTATAACCATGTGTAGAGTTTTTTTTTTTTTTTTTTTT - Biotin
T	GGATAACCTACCTATAAGACTGGGTTTTTTTTTTTTTTTTTTTT - Biotin

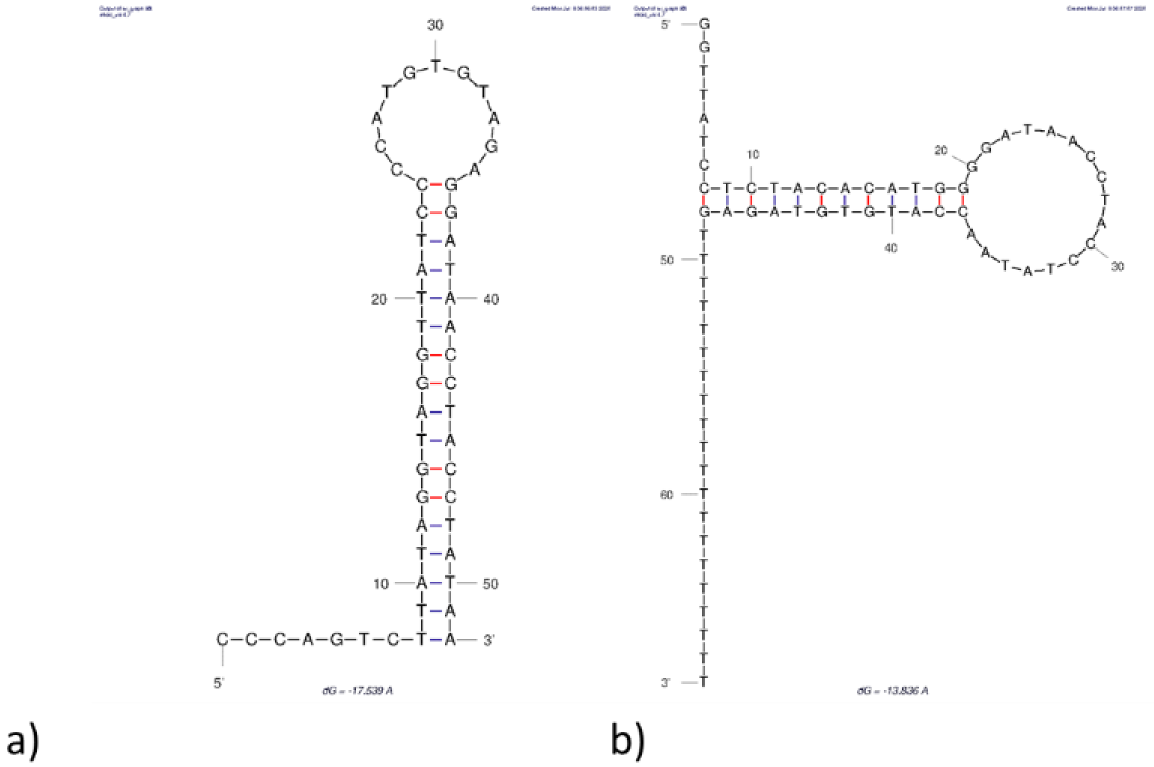

Figure S1 Predicted structure of hairpin DNA folding a) HA and b) HB given from Mfold web server.[1]

2. Assembly of a FPL composed of ssDNA

FPL was assembled from a pool of ssDNA oligos listed in **Table S2** and named as Stand 0 – 9 with additional three ssDNA oligos carrying T sequence conjugated via a poly(T20) linker to the Strand 0, 2 and 4 (marked as 0-T, 2-T, and 4-T). In order to assemble the FPL with different *NS*, a subset of strands specified in **Figure S2** were dissolved in the assembling buffer (e.g. for FPL with *NS*=5, strand2-T, 3, 4, 5, 6, and 7 were used). The mixture was heated to 95 °C for 5 min followed by colling to 25 °C at the rate of 1 °C per minute (see the detail in the Material and Methods section in the main manuscript). The assembled FPL was used for the experiments mentioned in the main manuscript without purification.

Table S2 Sequence of ssDNAs used to assembly FPL.

Name	Abbreviation	5′ → 3′
Strand0-T	0-T	GGATAACCTACCTATAAGACTGGGTTTTTTTTTTTTTTTTTTTTAAAAATCGCCCGCTCCTT AGCTTGTAACCGAAGGTCCCAACGCAT
Strand1	1	CTTGCACTGCGGGAGAGCTGCGTCGTTTGCTACAAGTCTCCATTCCTTATCGATGTTCGA GTGGATGGGGTACAAGGACCGGACCTATCA
Strand2	2	TACCGAGGGGTTAGCAGGTCCCGCGATGCCTGTACATCACGGTATAAGCGAACAACAAT AAATCCACAGGGCTGAAGACGCTGACGGGTT
Strand3	3	AACAGTTTTCCTACGTAATTCGCTGTGGCCAGGAAAGTTTCCACCATCACTGTTATTATGA GATAAGATTTCTTGCCCTAAGCAGAGGTG
Strand4	4	GCGACCGCGGCAGTCTATGTGAACTCATTGTTTTTCCACGATTTACGACAAAACAGGAA TATCGTCCACCCCAGTCAACCATGTGTAGAG
Strand5	5	Biotin - TTTTTTTTTTTTTTTTTTTTCTCTACACATGGTTGACTGGGGTGGACGATATTCCTGTTTTG TCG
Strand6	6	TAAATCGTGGAAAAACAATGAGTTCACATAGACTGCCGCGGTCGCCACCTCTGCTTAGG GCAAGAAATCTTATCTCATAATAACAGTGAT
Strand7	7	GGTGGAAACTTTCCTGGCCACAGCGAATTACGTAGGAAAACTGTTAACCCGTCAGCGTC TTCAGCCCTGTGGATTTATTGTTGTTCGCTT
Strand8	8	ATACCGTGATGTACAGGCATCGCGGGACCTGCTAACCCCTCGGTATGATAGGTCCGGTCC TTGTACCCCATCCACTCGAACATCGATAAG
Strand9	9	GAATGGAGACTTGTAGCAAACGACGCAGCTCTCCCGCAGTGCAAGATGCGTTGGGACC TTCGGTTACAAGCTAAGGAGCGGGCGATTTTT
Strand2-T	2-T	GGATAACCTACCTATAAGACTGGGTTTTTTTTTTTTTTTTTTTTAAGCGAACAACAATAAA TCCACAGGGCTGAAGACGCTGACGGGTT
Strand 4-T	4-T	GGATAACCTACCTATAAGACTGGGTTTTTTTTTTTTTTTTTTTTCGACAAAACAGGAATAT CGTCCACCCCAGTCAACCATGTGTAGAG

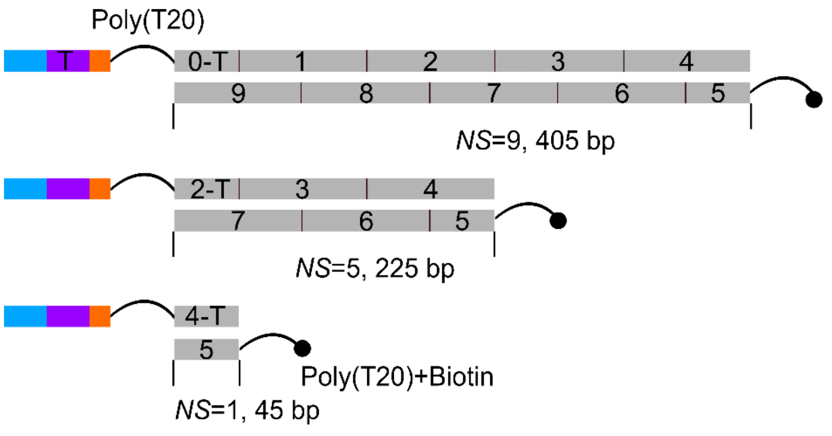

Figure S2 Combination of ssDNA strands to assemble FPL with segments *NS*=1, 5, and 9.

3. Prediction of average distance between NA molecules

The average distance between NA molecules *d* was obtained as:

$$d = \sqrt{A} = \sqrt{\frac{1}{n_{NA}}} = \sqrt{\frac{MW_{NA}}{\Gamma \cdot N_A}} \quad , \qquad (1)$$

where A is footprint area of the immobilized single NA molecule (units of nm^2), n_{NA} is the number of NA molecule per unit area (units of molecule/nm^2), Γ is the surface mass density of immobilized NA molecules (units of ng/mm^2), $N_A = 6 \times 10^{23}$ is Avogadro number (units of molecules/mol), and MW_{NA} is the molecular weight of NA molecule set to 60 kDa (units of g/mol). For the surface mass density of immobilized NA molecules Γ=4.14 ng/mm^2 (obtained from measured SPR response of 8.3 mRIU in **Figure S3**), the average distance was determined as *d*=4.9 nm.

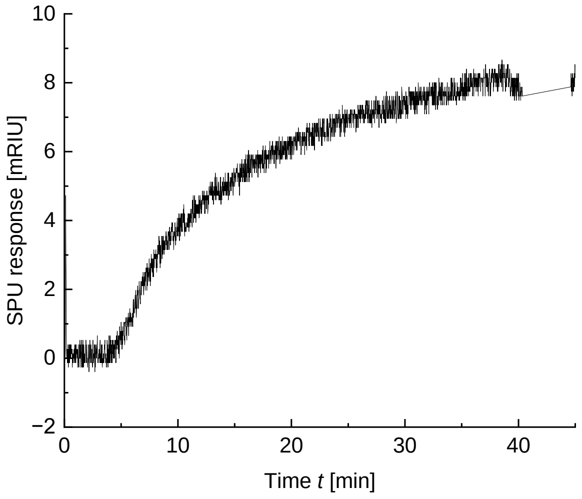

Figure S3 SPR response of immobilizing NA molecules in a sample liquid reacted with the biotin-coated gold surface

4. Prediction of the end-to-end distance of FPL

The end-to-end distance of FPL *r* was estimated from a probability function *P*(*r*):

$$P(r) = 4\pi r^2 (\frac{3}{2\pi NSa^2})^{3/2} e^{(\frac{-3r^2}{2NSa^2})} \ , \qquad (2)$$

where *NS* is the number of rigid segments and *a* is the length of these rigid segments. The simulated probability is plotted in **Figure S4** for two FPLs with *NS*=5 and 9.

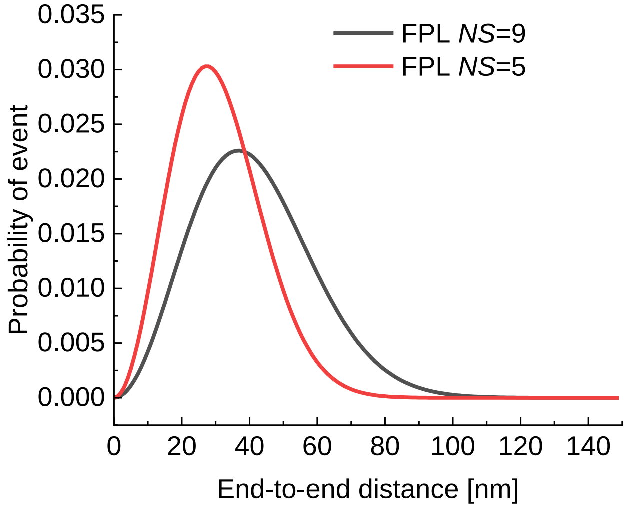

Figure S4 Probability distribution of the end-to-end distance for two FPLs given from the used mathematical model as probability function in Equation S2.

5. Time series of PEF images of tCHA

After tCHA was started by the injection of HA-Cy5, series of PEF images was acquired every 5 min by EMCCD camera with an exposure time of 10 seconds and accumulation of 6 times. Four representative examples are shown in **Figure S5** for to reaction time of *t* = 0, 30, 60, and 90 min when there was used FPL with *NS*=9 at *c*(T)=10 fM and *c*(HA)=10 nM.

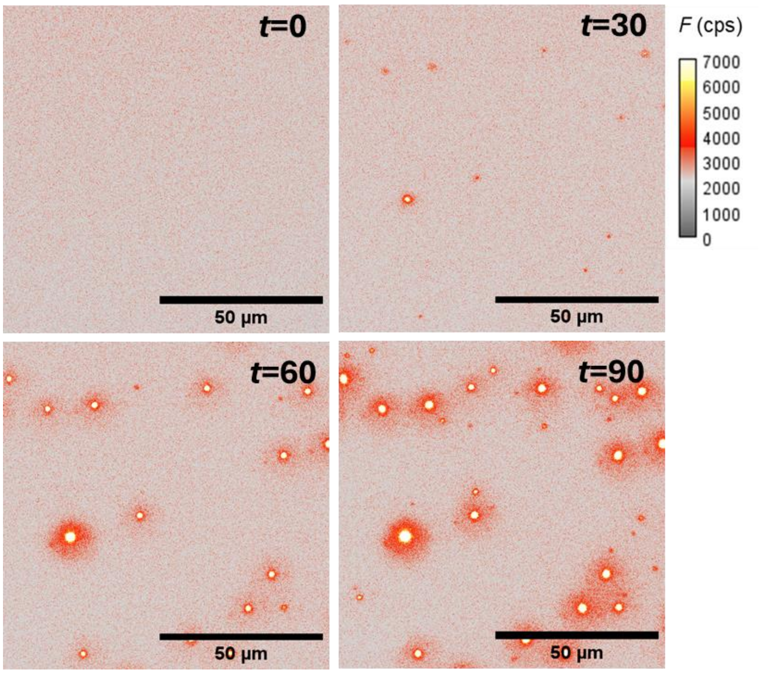

Figure S5 Time lapse PEF imaging of tCHA using FPL with *NS*=9 at *c*(T)= 10 fM and *c*(HA)= 10 nM).

6. Analysis of diffusion-limited analyte binding

An analysis of the number of captured solute molecules on the sensing surface can be estimated analytically by assuming that the affinity capture of solute (in the form of FPL-T being captured by NA, or later in the form of IL-6 being captured by cAb) is instantaneous and irreversible (i.e., diffusion-limited binding). The diffusion-limited transport of a solute flowing in a rectangular microchannel to a solid sensing surface (here, the floor of such microchannel) is best examined from a local perspective as a function of axial position (*z*).[2] Here, as the width of the microchannel (*W* = 1mm) is much smaller than its height (*H* = 0.05mm), we can safely model the system as 2D flow in a slot, where then the flux of solute to the bottom surface is then solely a function of axial distance $j_z = j_z(z)$. This flux can be made dimensionless at all axial positions as:

$$F = \frac{j_z H}{C_a D_a}, \quad (3)$$

where D_a is the diffusivity of the solute, and $C_a = C_a(z)$) is the "mixing-cup" concentration of analyte at axial position z (normalized in terms of molar flow rate), where z can be made dimensionless as:

$$\bar{z} = \frac{zD_a}{QWH}, \quad (4)$$

where Q is the volumetric flow rate of liquid through the channel. In this manner (no matter the value of Q, W, H, or D_a) the dimensionless flux will follow a trend that can be approximated by:

$$F = \left(\bar{z}^{-5/3} + 2.45^5\right)^{1/5}, \quad (5)$$

where the 1st term in the parenthesis is related to the diffusion of solute through a thin boundary layer (whose thickness slowly grows along z), and the 2nd term concerns a phase such that the boundary layer has enveloped the entire height of the microchannel (the value 2.45 is the asymptotic Sherwood number). In this case C_a can be calculated along the entire length of the channel as:

$$C_a(z) = C_o \exp\left(-\int_0^z \frac{FD_a}{QWH} dz\right), \quad (6)$$

where it follows that C_o is the concentration of the injected solute. To solve for the solute flux at a channel length L, Eqn. (S6) is integrated using both Eqn. (S5) for values of F, where the integration is carried out at $z = L$, (where D_a, Q, W, H, and C_o are taken from experimental values) to obtain $C_a(L)$, which along with $F(L)$, can then be used to directly calculate the solute flux at an axial position L using Eqn. (S3).

Table S3 Parameters used to estimate solute transport to the sensing surface. The diffusion coefficients were based on modification of the experimental measurements of bovine serum albumin (D_a = 6×10^{-5} mm^2/s, Mw = 66 kDa [3]), where we assumed diffusivities scaled with the inverse cube of molecular weight.

Description	Parameter	Value
geometrical parameters		
Channel height	H	50 µm
Channel width	W	1 mm
Axial length to analysis region	L	21 mm
Operational parameters		
Diffusivity of IL6 (20 kDa)	D_a	8.9×10^{-5} mm^2/s
Diffusivity of HB (21 kDa)	D_a	1×10^{-4} mm^2/s
Diffusivity of FPL-T (2.7 MDa)	D_a	1.7×10^{-5} mm^2/s
Volumetric flow rate (each inlet)	Q	10 µL/min

Estimation of HB and FPL-T transport to the sensor surface. A schematic of the channel outline for the PEF sensor is shown in the inset in **Figure. S6a**. An estimated length of 21mm is sufficient for all the 4 sensing microchannels. Using this length and the other information in Table S3, we can use the analytical method shown to calculate the molecular flux along the axial length of the channels, from which the molecular capture rate can be estimated using the 0.01 mm PEF footprint. Figure S6a plots the molecular capture rate for both HB and FPL-T along the entire length of the channel, which further shows why the methodology shown above is necessary, as both the localized capture rate near the inlet and the average rate of capture along the entire channel (for both HB and FPL-T) are significantly higher than the region of channel in the PEF imaging zone. Estimates of the HB molecular capture rates are approximately 2.6×10^5 higher than that of FPL-T along the entire length of the channel; the added difference of 2.6× from the 1:10^5 ratio of concentrations is due to the large difference in diffusivity between the two species.

Assuming a single NA/biotin interaction for each NA molecule (the others prohibited via stearic reasons), a 4.9 nm distance between NA molecules corresponds to approximately 4.1×10^8 binding sites within the 100×100 µm^2 PEF imaging area. The estimated flux values for both

HB (1 nM) and FPL-T (10 fM) correspond to a total of 4.2×10^7 and 96 captured molecules, respectively (for a 30 minute assay). These values are approximately 10× less than the total estimated number of binding sites, hence the diffusion-limited approach can be considered to be free from any limiting effects due to equilibrium. A similar approach can be taken to estimate the number of captured FPL-T molecules at variable concentration $c(T)$, the results of which can be seen in **Figure. S6b**. These estimates are plotted alongside the experimental values (taken from Figure. 6), where the majority of experimental data falls within a factor of 3× from the diffusion-limited estimate.

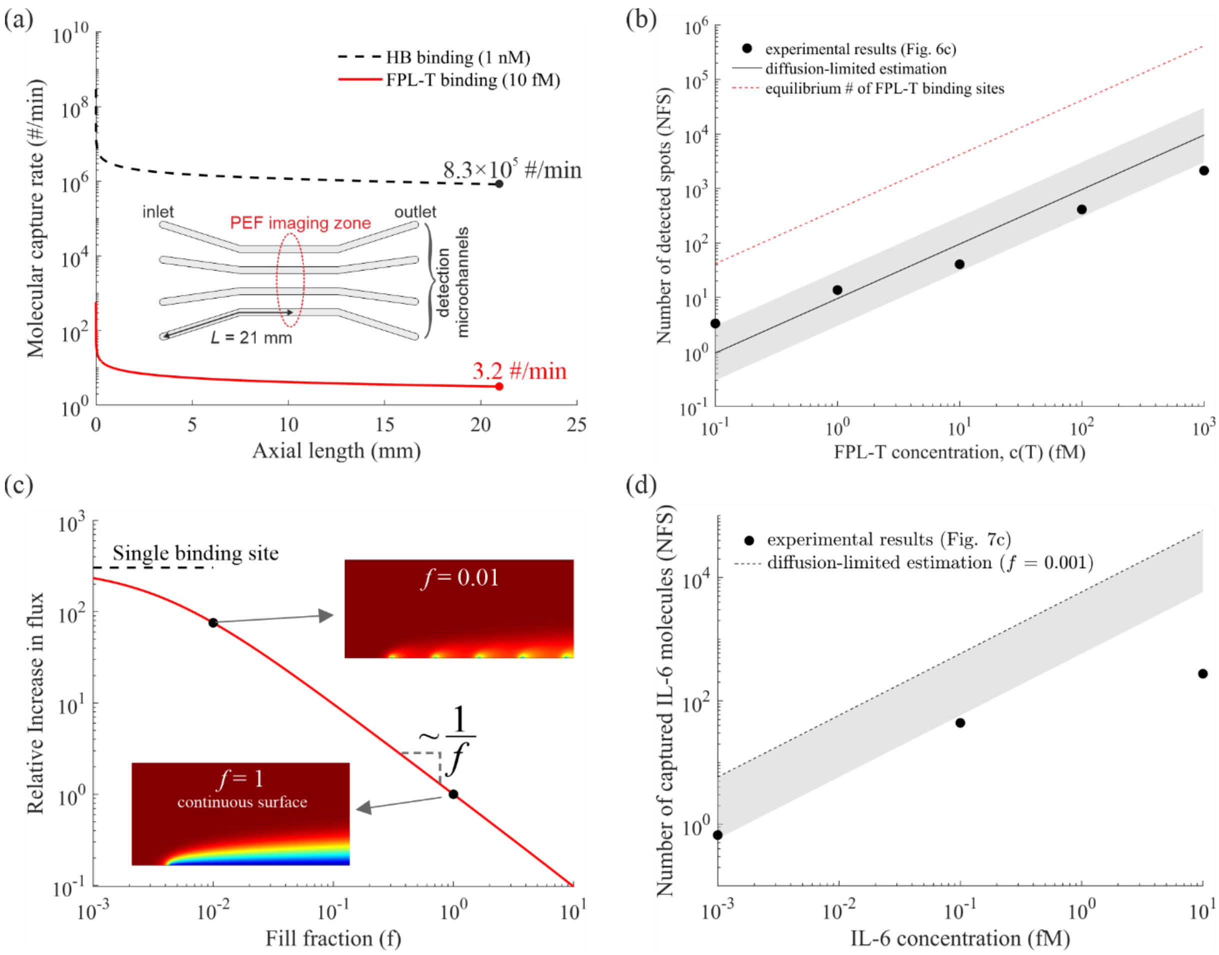

Figure S6. (a) Estimates of the molecular capture rate of both HB and FPL-T along the length of the microchannels pertaining to the experimental conditions described in Figure. 6 (given in **Table S3**). (b) Estimated number of captured FPL-T molecules (manifested experimentally as the number of detected spots (NFS)) as a function of FPL-T concentration. The shaded region represents ±3.2× on each side (representing a value of 10× overall), and the red dashed line represents the total number of binding sites for FPL-T at equilibrium (assuming a HB concentration of 1nM). (c) Using the methods shown in [4], we can estimate the relative

increase in solute flux to a surface according to the fill fraction. The insets show the qualitative shape of a solute depletion layer at both f = 1 (a continuous layer) and f =0.01. (d) Estimated number of captured IL-6 molecules, where the dashed line represents a 234× increase in flux with respect to a continuous surface (via [4]) due to the low fill fraction (f = 0.001) that is due to a lower concentration of cAb with respect to the HB molecules. The shaded region represents a 10× lower change from the dashed line.

To estimate the number of IL6 molecules captured we must make a slight modification to the analytical method described above. We first note that the capture of both HB and FPL-T is done under conditions where the available NA binding sites are dense enough (i.e., approaching a monolayer) such that we can assume a continuous layer of capture sites for diffusion-limited transport. The capture of IL-6, however, is done under significantly different circumstances: the preparation of the surface was carried out at a 1:10^3 (cAb:HB) concentration ratio, which pertains to an average distance between cAb molecules of d =155 nm (compared to an average distance between NA sites of d =4.9 nm). Due to the limited cAb surface coverage, we must modify the analytical approach above via the methods shown in the literature[4], where we assume that rather than a continuous capture surface, the detection surface is composed of an array of sparse cAb binding sites, each having the same footprint as the underlying NA molecule (diameter 4.9 nm). In this approach we first estimate the fill fraction (f) for cAb sites (i.e., the area for cAb binding with respect to the overall sensing area) as $f = (4.9\ \text{nm}/155\ \text{nm})^2 = 0.001$. The dependence of the relative increase in flux on f is shown in **Figure S6c** (calculated according to the literature[4]), where for the detection conditions specified in Table S3 we estimate a IL-6 flux increase of 234× (regardless of the concentration). These estimations are plotted along with the experimental data in **Figure S6d**, where it can be seen that this analytical method overestimates the number of captured IL-6 molecules by an order of magnitude. This overestimation is likely due to a number of factors, including induced inactivity of both cAb and dAb as a result of biotinylation, and as noted above, non-equilibrium conditions of IL-6 (as well as cAb) binding.

7. Control experiment for immunosandwich-tCHA assay

Figure S7 compares an example of PEF images acquired for IL6 sandwich immunoassay with tCHA readout when all assay constituents are included int the reaction and a control where the FPL was not reacted with the surface. The procedure followed the one described in the main text.

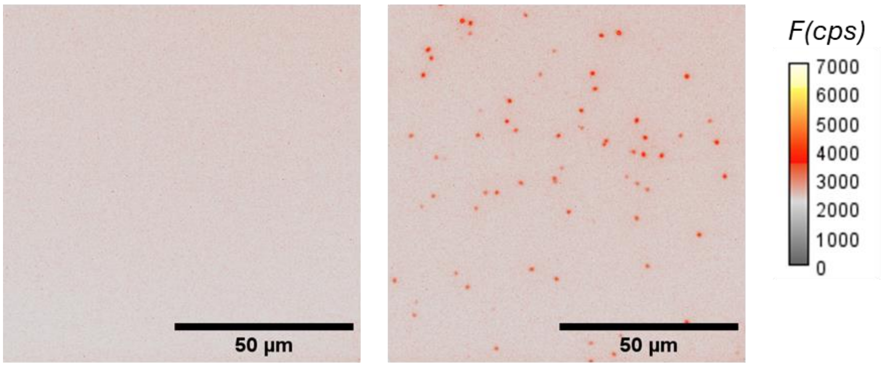

cAb-biotin (1 pM)<br>HB-biotin (1 nM)	+
IL6 (10 fM)	+
dAb+S5 (1 µg/mL)	+
FPL (1 nM)	–

cAb-biotin (1 pM)<br>HB-biotin (1 nM)	+
IL6 (10 fM)	+
dAb+S5 (1 µg/mL)	+
FPL (1 nM)	+

Figure S7 PEF images for control experiment where symbol + means that the assay component was included and – means its absence in the assay. FPL N=9 served for these assays. A solution containing c(HA-Cy5)=10 nM was flowed for 120 min and the PEF image was acquired following the protocol described in the main text.

8. Comparison of benchmarks on SMD and digital assay

Table S4 provides an overview of representative examples of digital assay recently reported in literature together with a comparison of their sensing performance.

Table S4 Overview of representative examples of recently reported digital assay relying on enzymatic and non-enzymatic reactions with and without partitioning of analyzed liquid sample.

Entry	LoD	Reaction time	Upper detection range	Target	Use of enzyme	Compartment	Microscopy	Refe renc e
Digital tCHA-PEF	0.12 fM	3-4 h	1 pM	ssDNA or IL6	No enzyme	Compartment-free affinity capture	ATR-Kretschmann configuration with wide field microscopy	This wor k
Digital assay with on chip pre-concentration	0.089 fM	40 min	n.a.	SARS-CoV-2 RNA	Cas13	Microwell with preconcentratio n	Laser scanning confocal fluorescence microscopy	[5]
Digital SERS	0.71 fM	5 h	37 nM	SARS-CoV-2 spike protein	No enzyme	Compartment-free affinity capture	Raman microscopy	[6]
Digital immunoassay with PEF labels	13 fM	4.5 h	230.4 pM	IL6	No enzyme	Compartment-free affinity capture	Wide field microscopy	[7]
Multiplexed digital droplet ELISA	0.13 fM	1 h	n.a.	IL6	HRP	Microdroplets	Wide field microscopy	[8]
Digital immunoassay with fluorescent microspheres	276.5 fM	15 min	2.3 pM	IL6	No enzyme	Compartment-free affinity capture	Wide field microscopy	[9]
Digital CHA-PEF	0.83 fM	2 h	1 pM	HIV-RNA	No enzyme	Compartment-free affinity capture	TIRF microscope	[10]
Digital RCA-PEF	4.3 fM	5 h	143 pM	IL 6	Polymera se	Compartment-free affinity capture	Laser scanning confocal fluorescence microscopy	[11]

9. Characterization of the deposited metal layers

Angular SPR reflectivity spectrum from used thin film structure were measured and analyzed based on Fresnel reflectivity model with transfer matrix formalism. WinSpal software (developed at Max Planck Institute for Polymer Research, Mainz, Germany) was used to fit the measured curves and the yield thickness *t* of the layers are *t*(Cr)=2.6 nm and *t*(Au)=50.6 nm.

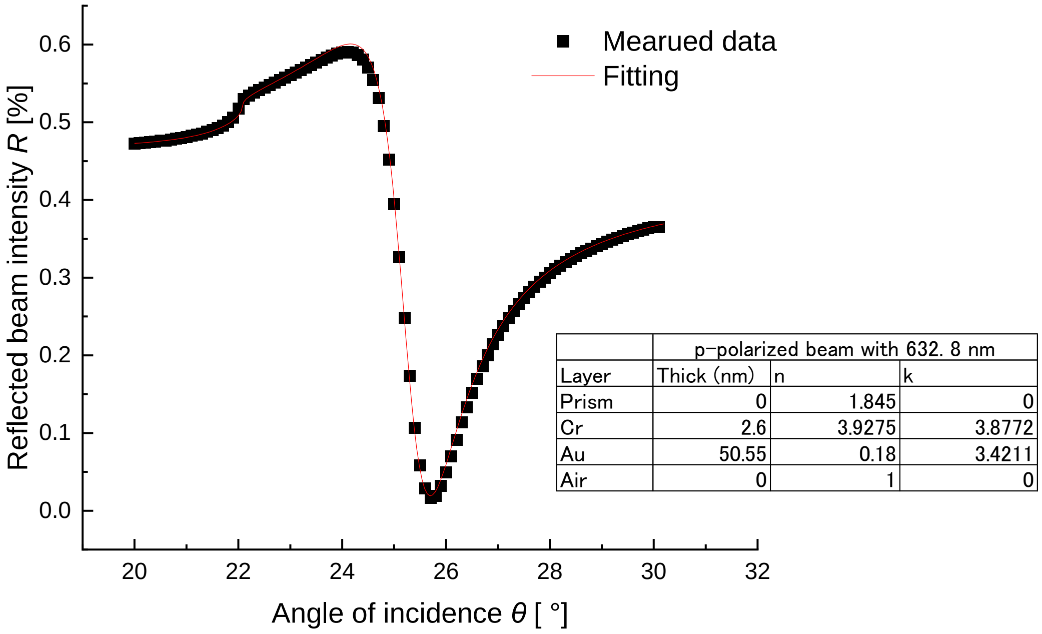

Figure S8 Angular reflectivity spectra of Au/Cr/glass substrate measured in air phase. Fitting was performed with the built-in WinSpal function.

10. Additional characterization of cAb and HB immobilization

The immobilization of cAb and HB was examined by using SPR biosensor and the coupling mechanism *via* biotin tags. The immobilization was tested at higher concentrations *c*(cAb-biotin)=166.67 nM and *c*(HB-biotin)=50 nM as can be seen in **Figure S9**.

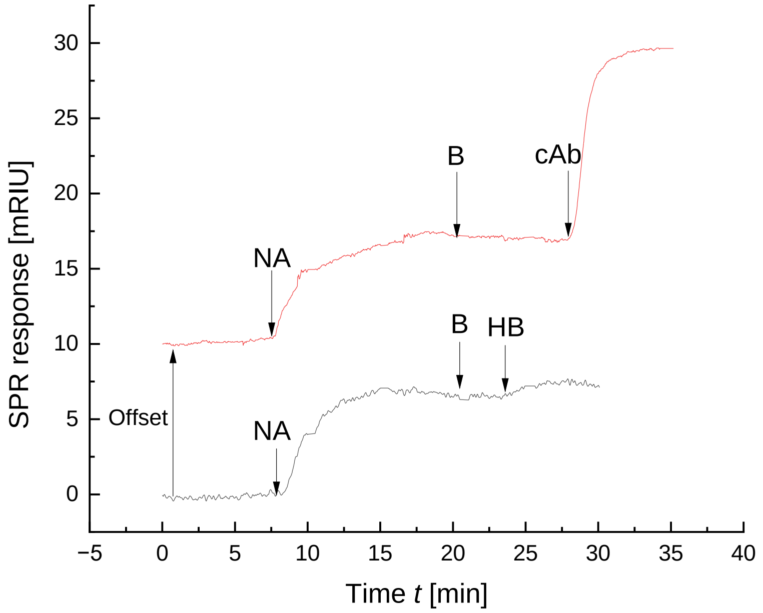

Figure S9 Kinetic reflectivity measurement of cAb-biotin and HB-biotin immobilization at concentrations c = 166.67 nM and 50 nM, respectively. The concentration of NA is c(NA)= 100 µg/mL. A black signal shows HB-biotin binding to the NA-immobilized interface. A red signal (cAb-biotin) is offset + 10 mRIU for clear visibility. B indicates washing process with PBST.

11. References

[1] M. Zuker, *Nucleic Acids Res.* **2003**, *31*, 3406.
[2] N. S. Lynn Jr, in *Integrated Analytical Systems*, Springer International Publishing, Cham, **2018**, pp. 69–103.
[3] A. K. Gaigalas, J. B. Hubbard, M. McCurley, S. Woo, *J. Phys. Chem.* **1992**, *96*, 2355.
[4] N. S. Lynn Jr, J. Homola, *Anal. Chem.* **2016**, *88*, 12145.
[5] Y. Minagawa, S. Nakata, M. Date, Y. Ii, H. Noji, *ACS Nano* **2023**, *17*, 212.
[6] J.-E. Shim, Y. J. Kim, J.-H. Choe, T. G. Lee, E.-A. You, *ACS Appl. Mater. Interfaces* **2022**, *14*, 38459.
[7] A. Seth, Y. Liu, R. Gupta, Z. Wang, E. Mittal, S. Kolla, P. Rathi, P. Gupta, B. A. Parikh, G. M. Genin, S. Gandra, G. A. Storch, J. A. Philips, I. A. George, S. Singamaneni, *Nano Lett.* **2024**, *24*, 229.
[8] J. Yi, Z. Gao, Q. Guo, Y. Wu, T. Sun, Y. Wang, H. Zhou, H. Gu, J. Zhao, H. Xu, *Sens. Actuators B Chem.* **2022**, *369*, 132214.
[9] Y. Xie, H. Li, Y. Tang, X. Lian, L. Dai, S. Tian, *Sens. Actuators B Chem.* **2023**, *396*, 134547.
[10] K. Shi, N. Na, J. Ouyang, *Analyst* **2022**, *147*, 604.
[11] K. Schmidt, T. Riedel, A. de los Santos Pereira, N. S. Lynn, D. F. Dorado Daza, J. Dostalek, *ACS Appl. Mater. Interfaces* **2024**, *16*, 17109.
